# Identification of genes related to agarwood formation: transcriptome analysis of healthy and wounded tissues of *Aquilaria sinensis*

**DOI:** 10.1186/1471-2164-14-227

**Published:** 2013-04-08

**Authors:** Yanhong Xu, Zheng Zhang, Mengxi Wang, Jianhe Wei, Hongjiang Chen, Zhihui Gao, Chun Sui, Hongmei Luo, Xingli Zhang, Yun Yang, Hui Meng, Wenlan Li

**Affiliations:** 1 Institute of Medicinal Plant Development, Chinese Academy of Medical Sciences & Peking Union Medical College, No. 151, Malianwa North Road, Haidian District, Beijing, 100193, China; 2 Hainan Branch Institute of Medicinal Plant (Hainan Provincial Key Laboratory of Resources Conservation and Development of Southern Medicine), Chinese Academy of Medical Sciences & Peking Union Medical College, Wanning, 571533, China; 3 Research Center on Life Sciences and Environmental Sciences, Harbin University of Commerce, Harbin, 150076, China

**Keywords:** Agarwood, *Aquilaria sinensis*, GC-MS, Sesquiterpenes, Transcriptome, Wound signal transduction

## Abstract

**Background:**

Agarwood is an expensive resinous heartwood derived from *Aquilaria* plants that is widely used in traditional medicines, incense and perfume. Only wounded trees can produce agarwood, and the huge demand for the agarwood products has led all *Aquilaria* spp. being endangered and listed in the Appendix II of the CITES (http://www.cites.org). The major components of agarwood are sesquiterpenes and phenylethyl chromones. Owing to a lack of genomic information, the molecular basis of wound-induced sesquiterpenes biosynthesis and agarwood formation remains unknown.

**Results:**

To identify the primary genes that maybe related to agarwood formation, we sequenced 2 cDNA libraries generated from healthy and wounded *A. sinensis* (Lour.) Gilg. A total of 89,137 unigenes with an average length of 678.65 bp were obtained, and they were annotated in detail at bioinformatics levels. Of those associated with agarwood formation, 30 putatively encoded enzymes in the sesquiterpene biosynthesis pathway, and a handful of transcription factors and protein kinases were related to wound signal transduction. Three full-length cDNAs of sesquiterpene synthases (*ASS1-3*) were cloned and expressed in *Escherichia coli*, and enzyme assays revealed that they are active enzymes, with the major products being δ-guaiene. A methyl jasmonate (MJ) induction experiment revealed that the expression of *ASS* was significantly induced by MJ, and the production of sesquiterpenes was elevated accordingly. The expression of some transcription factors and protein kinases, especially *MYB4*, *WRKY4*, *MPKK2* and *MAPK2*, was also induced by MJ and coordinated with *ASS* expression, suggesting they maybe positive regulators of *ASS*.

**Conclusions:**

This study provides extensive transcriptome information for *Aquilaria* spp*.* and valuable clues for elucidating the mechanism of wound-induced agarwood sesquiterpenes biosynthesis and their regulation.

## Background

Agarwood is widely used in traditional medicines as a digestive, sedative, and antiemetic drug and is also popular as incense and perfume in the Middle East, South Asia, Japan, and China. Additionally, the agarwood sculpturing for interior decoration is another important aspect of its value which generates a lot of income in Asia. In the international market, high-quality agarwood is more costly than gold. Agarwood is a dark, resinous, non-timber wood that forms in the stem, branch, or root of *Aquilaria* and *Gyrinops* trees after they are wounded and infected by a fungus (under natural conditions, the wounds can be caused by wind, lightning strikes, the gnawing of ants or insects, or microorganism invasion), but these natural processes always develop very slowly over decades. Owing to the economic value and great demand of agarwood, natural *Aquilaria* forests have been destroyed in almost all countries in which agarwood is commercially exploited. For the protection of wild *Aquilaria* resources and their sustainable use, all *Aquilaria* spp. have been listed in Appendix II of the Convention on International Trade in Endangered Species of Wild Fauna and Flora (http://www.cites.org [accessed 7 August 2012]), and *Aquilaria* cultivation has been drawn much attention in countries such as China, India, Vietnam, Indonesia, Malaysia, and Thailand. However, the current methods used by farmers, including partial trunk pruning, burn-chisel-drill, and fungi inoculation, all require considerable time to produce agarwood and result in a product with very low yield and quality.

*Aquilaria sinensis* (Lour.) Gilg is one of the most important plant resources for producing agarwood in China as well as the only certified source for agarwood listed in *China Pharmacopoeia*[1]. More than 20 million planted *A. sinensis* trees are estimated to be scattered throughout Hainan, Guangdong, and Yunnan Provinces, and more than one-fourth of them are older than 5 years and have become available for agarwood induction. Uncovering the mechanism of agarwood formation in wounded trees is crucial to establishing an efficient induction method for agarwood.

Studies have shown that sesquiterpenes and phenylethyl chromone derivatives are the main compounds in agarwood [2-7]. Thus, understanding the biosynthesis and regulation of sesquiterpenes and chromone in *Aquilaria* spp. is critically important in determining the mechanism of agarwood formation. However, to date, the biosynthesis pathway of chromone derivatives remains almost unknown. The terpenoid metabolism pathway is, by comparison, very clear. The biosynthesis of sesquiterpenes can reportedly occur via the mevalonic acid (MVA) [8,9] and 1-deoxy-d-xylulose-5-phosphate (DXP) [10] pathways (Additional file 1: Figure S1), in which sesquiterpene synthases are enzymes used in the final step to form sesquiterpenes. Currently, many studies on the functional identification and regulation of these enzymes have been reported. In *Gossypium arboreum*, GaWRKY1 positively regulates the expression of (+)-δ-cadinene synthase, which catalyzes the branch point leading to biosynthesis of sesquiterpene gossypol [11]. AaWRKY1 regulates the expression of amorpha-4,11-diene synthase (ADS), which is the key artemisinin biosynthesis enzyme in *Artemisia annua*. Overexpression of AaWRKY1 in tobacco or in *A. annua* can significantly activate ADS promoter activity, and artemisinin production increases accordingly [12]. The latest report on *Arabidopsis* has shown that transcription factor (TF) MYC2 interacts with DELLA proteins, positively regulating the expression of sesquiterpene synthases TPS21 and TPS11 in the jasmonic acid (JA) and gibberellic acid (GA) signaling pathways [13]. However, studies on agarwood sesquiterpenes in *Aquilaria* spp*.* are still extremely limited, and with the exception of five δ-guaiene synthase genes (*AcC1*, *AcC2*, *AcC3*, *AcC4*, and *AcL154*), which were cloned from cell cultures of *A. crassna*[5], most of the genes remain to be cloned. Kumeta & Ito [5] have also found that methyl jasmonate (MJ) treatment increases the expression of these genes and induces the production of sesquiterpene δ-guaiene. Their report was the first and remains the only molecular report of sesquiterpene biosynthesis and regulation in *Aquilaria* spp..

Agarwood is produced after an *Aquilaria* tree is wounded [14-17]. We have observed that in the early period of agarwood formation, the resin in agarwood exists in 2 forms: tylose and gel (our unpublished data), and the sesquiterpenes in agarwood have antimicrobial and anti-disease activity [6]. Based on previous studies, we have hypothesized that agarwood is the product of a plant defense response [18]: when an *Aquilaria* tree is wounded, damage signals are induced and transmitted, activating the defense response, after which defensive substances such as sesquiterpenes and phenylethyl chromone derivatives are produced. When these products combine with the wood tissue to avoid damage expansion, agarwood forms. The key to testing this hypothesis is to reveal the mechanism of wound-induced biosynthesis of agarwood sesquiterpenes, but this goal is hindered by the very limited amount of genomic information available for *Aquilaria*. The present study made use of 454 sequencing technology in order to (i) provide detailed insight into the transcriptomes of healthy and wounded *A. sinensis*, (ii) discover candidate transcripts with significant homology to important enzymes in the sesquiterpene biosynthesis pathway and verify their function, and (iii) identify candidate regulators that might be related to wound signal transduction and regulation of agarwood formation.

## Results

### Transcriptome sequence assembly and annotation

**Figure 1:**
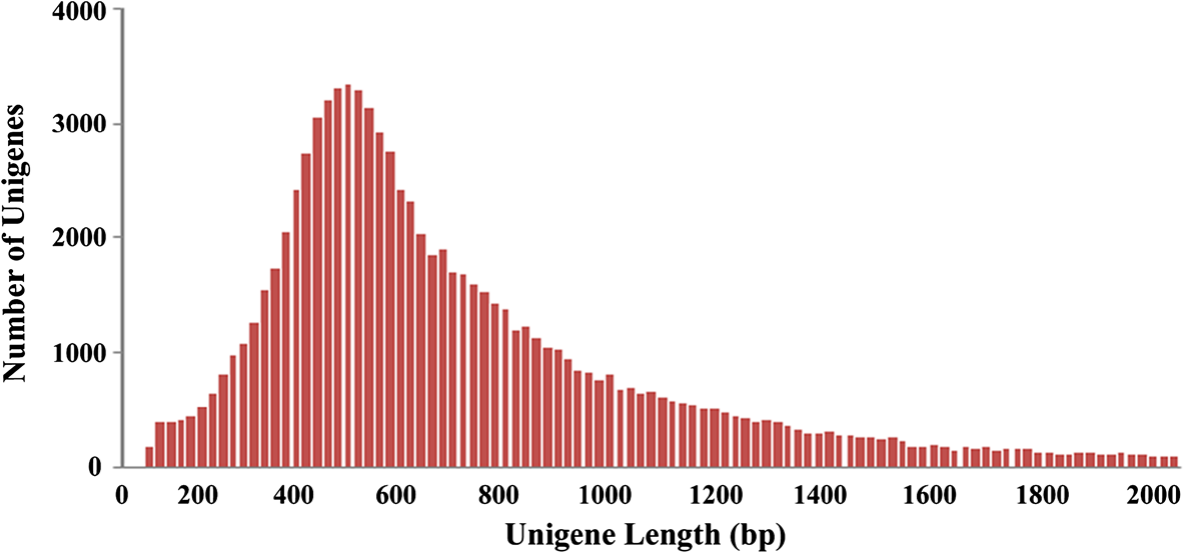
**Length distribution for *Aquilaria sinensis *unigenes.** Over 70% of these unigenes are between 200 bp and 600 bp long.

Two cDNA libraries from the stems of the healthy and wounded *A. sinensis* (designated H and W) were constructed using Switching Mechanism At 5' end of RNA Transcript (SMART) technology. The 2 libraries were sequenced in one run using 454 GS-FLX pyrosequencing technology. A total of 562,060 (H) and 565,008 (W) raw reads were generated (Table 1). After the removal of sequences shorter than 100 bp and empty vectors, 1,073,469 high-quality reads [NCBI GEO, accession no. GSE42246], including 530,438 (94.37% of the raw reads) from the H library and 543,031 (96.11% of the raw reads) from the W library, were obtained. After *de novo* assembly of these reads, 67,972 unigenes from the H library and 74,139 unigenes from the W library were generated, respectively (Table 1). Combining the reads from the 2 libraries, we obtained 89,137 unigenes with an average length of 678.65 bp. More than 70% of these unigenes were between 200 bp and 600 bp long (Figure 1), and almost 17% contained more than 10 reads (Additional file 2: Table S1).

**Table 1:** **Characteristics of the reads from the healthy and the wounded *A*. *sinensis***

**Numbers**	**Healthy**	**Wounded**
Total reads	562,060	565,008
Vector reads^a^	2,698	2,095
Low quality reads^b^	28,924	19,882
High quality reads^c^	530,438	543,031
Contigs^d^	66,207	71,657
Singlets^e^	1,765	2,482
Unigenes^f^	67,972	74,139

^a^ Vector reads: The reads sequences are all vector sequences.

^b^ Low quality reads: After removing empty reads and vector reads, the length of reads is less than the cutoff length (100 bp).

^c^ High quality reads: After removing empty reads and vector reads, the length of reads is more than the cutoff length (100 bp).

^d^ Contigs: reads number ≥ 2.

^e^ Singlets: reads = 1.

^f^ Unigenes = Contigs + Singlets.

The BLAST annotation of the 89,137 unigenes indicated that 22,051 (24.7%) shared homology with the sequences in the NCBI nucleotide database, and 38,159 (42.8%) had matches to known protein sequences in the NCBI non-redundant database. To obtain more information, we also carried out the BLASTX search against the SWISS-PROT protein database, and 17.1% of the unigenes were thus annotated (Additional file 3: Table S2), indicating that the information about the genomes and transcriptomes of *Aquilaria* spp*.* was very limited. Thus, our 454 database is important, and the unigene sequences obtained in this study may represent most of the novel genes of *Aquilaria* spp..

**Figure 2:**
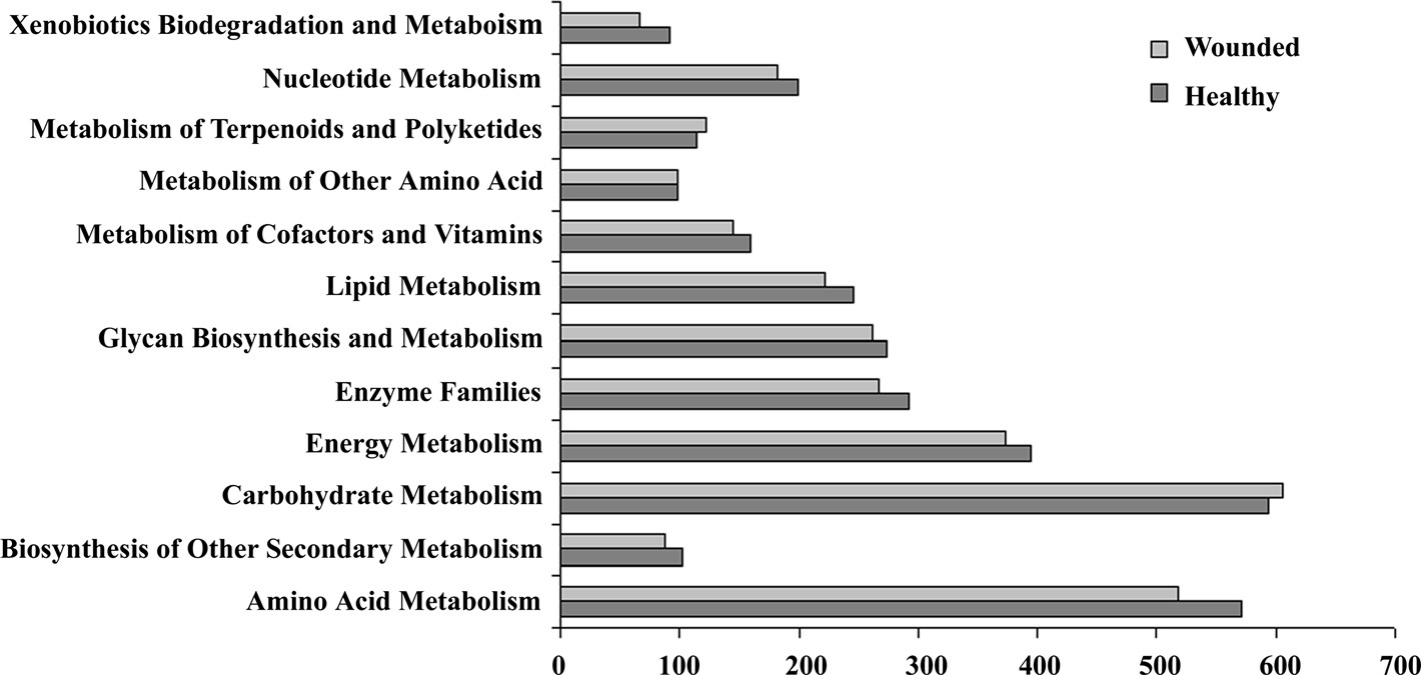
Comparison of KEGG metabolism categories in the wounded and healthy tissue libraries.

Based on an InterPro analysis, 48,530 (54%) unigenes were annotated with at least one GO term, and the distribution of gene functions based on the cellular component, molecular function, and biological process categories was very similar in the 2 libraries (Additional file 4: Figure S2). KEGG annotation showed that, in the metabolism process, although the W library had a total number of unigenes smaller than that of the H library, it had a slightly higher number of unigenes related to carbohydrate metabolism and metabolism of terpenoids and polyketides (Figure 2). This result indicated that the pruning wound in *A. sinensis* had activated carbohydrate, terpenoid, and polyketides metabolism, which may be related to agarwood formation.

**Figure 3:**
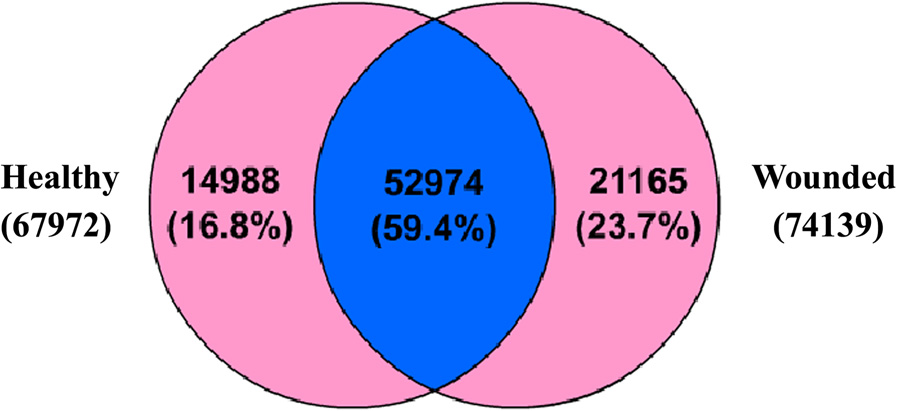
**Comparison of the unigenes expressed in the healthy and wounded tissue libraries.** The overlapping section represents the 52,974 (59.43%) unigenes that were found in both libraries; 14,988 (16.83%) unigenes were expressed only in the healthy library, and 21,165 (23.74%) unigenes were expressed only in the wounded library.

**Figure 4:**
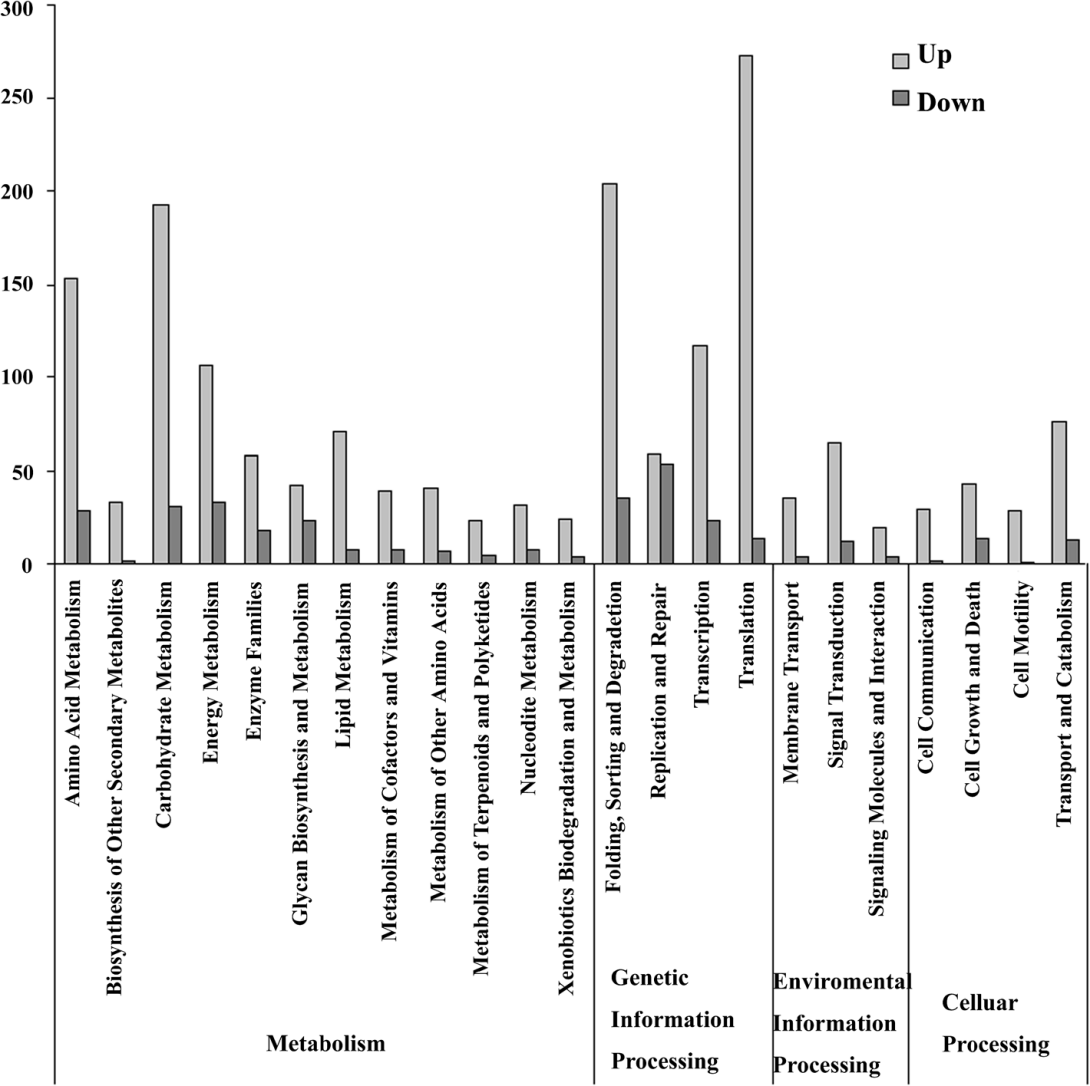
**Functional classification of differentially expressed genes based on KEGG classification.** In the categories metabolism, genetic information processing, environmental information processing, and cellular processing, the number of “up” unigenes was far greater than the number of “down” unigenes (the relative abundance of wounded library vs. healthy library >1 marked “up”, <1 marked “down”).

To investigate the differences between the 2 libraries, IDEG6 [19] was used to identify differentially expressed genes (DEGs). A total of 47,169 DEGs were obtained, including 14,988 and 21,165 expressed uniquely in the H and W libraries, respectively; 11,016 were expressed in both libraries but at different levels (Figure 3). KEGG classification showed that the number of “up” unigenes was far greater than the number of “down” unigenes (the relative abundance of the W library vs. the H library: >1 marked “up”, <1 marked “down”; Figure 4): for metabolism and genetic information processing, 814 (82.5%) and 652 (83.8%) of the DEGs were “up”, respectively. The percentage and distribution of DEGs annotated with GO terms are shown in Additional file 5: Figure S3.

### Enzyme genes involved in sesquiterpenes biosynthesis

The biosynthesis of sesquiterpenes occurs via the MVA [8,9] and DXP [10] pathways, and 4 key enzymes are commonly involved: 3-hydroxy-3-methylglutaryl-coenzyme A reductase (HMGR), the first key enzyme in the MVP pathway; DXP synthase (DXPS), the first rate-limiting enzyme in the DXP pathway; farnesyl diphosphate (FPP) synthase (FPS); and sesquiterpenes synthases. In our 454 unigene database (H + W), 30 unigenes were annotated as being related to the sesquiterpene biosynthesis pathway, and 22 of them putatively encode these enzymes, including 3 HMGRs, 12 DXPSs, 2 FPSs, and 5 sesquiterpene synthases (Table 2). Unigenes putatively encoding other enzymes in the sesquiterpene biosynthesis pathway were also found, including acetyl-coenzyme A acyltransferase (ACTC), mevalonate kinase (MK), phosphomevalonate kinase (PMK), and 2-C-methyl-d-erythritol-2, 4-cyclodiphosphate synthase (MCS) (see Table 2).

**Table 2:** Number of unigenes and 454 annotated reads involved in sesquiterpenes biosynthesis

**Enzyme Code (EC)**	**Abbreviated enzyme name**	**Enzyme name**	**Numbers of unigenes**	**Numbers of 454 reads**	
				**Healthy**	**Wounded**
2.3.1.16	ACTC	acetyl-CoA acyltransferase	2	19	0
1.1.1.34	HMGR	HMG CoA reductase	3	151	21
2.2.1.7	DXPS	1-deoxy-D-xylulose-5- phosphate synthase	12	35	37
1.1.1.267	DXPR	1-deoxy-D-xylulose-5- phosphate reductoisomerase	1	3	2
2.7.1.36	MK	mevalonate kinase	1	48	1
2.7.4.2	PMK	phosphomevalonate kinase	3	9	12
4.6.1.12	MCS	2-C-methyl-D-erythritol 2,4-cyclodiphosphate synthase	1	11	11
2.5.1.10	FPS	farnesyl diphosphate synthase	2	78	5
	SS	sesquitrpene synthase	5	383	10

FPS is a branch point enzyme in terpenoid biosynthesis [20-22]. Its product, FPP, is the common precursor of sesquiterpenes, steroids, and farnesylated proteins. We found two unigenes that putatively encode FPS. One is a full-length cDNA (named *AsFPS*) that has an open reading frame of 1029 nucleotides and encodes a protein of 342 amino acids. The phenetic relationship between *AsFPS* and the FPSs characterized in other species is depicted in Additional file 6: Figure S4. *AsFPS* had the highest homology with the FPS sequence from *Aquilaria. microcarpa* (GenBank ID: ADH95185; 97% identity, with a difference of 5 amino acids), suggesting that *AsFPS* belongs to the FPS family.

With the exception of one FPS, the full-length open-reading frames (ORFs) of other unigenes were unavailable. Sesquiterpene synthases are the key enzymes that catalyze FPP, leading to sesquiterpenes biosynthesis. Therefore, they were the focus of this study.

### Cloning and functional identification of *A. quilaria* sesquiterpene synthase genes (*ASSs*)

Based on the degenerated primers, 3 full-length cDNAs of sesquiterpenes synthases (*ASS1*, *ASS2*, and *ASS3*) were cloned and deposited in the NCBI database under the accession numbers JQ712682, JQ712683, and JQ712684. They have ORFs of 1644 nucleotides, which encode almost the same protein of 547 amino acids. Their nucleotide and protein sequences have more than 92% identity with one another. The alignment of their nucleotide and deduced amino acid sequences are shown in Additional file 7: Figures S5 and Additional file 8: Figures S6, respectively. These proteins contain 2 motifs (the RRx_8_W motif at the N-terminus and the DDxxD motif known to be a divalent metal ion substrate-binding site) that are functionally important and highly conserved in all terpene synthase proteins. The phenetic relationship between the 3 putative proteins and other characterized sesquiterpenes synthases is shown in Additional file 9: Figure S7. ASS1-3 were grouped with ACY38194, ACY38195, ACY38196, ACY38197 [5], AEG77018, AEG77021, and AEG77019 [23], which were cloned from *A. crassna*.

**Figure 5:**
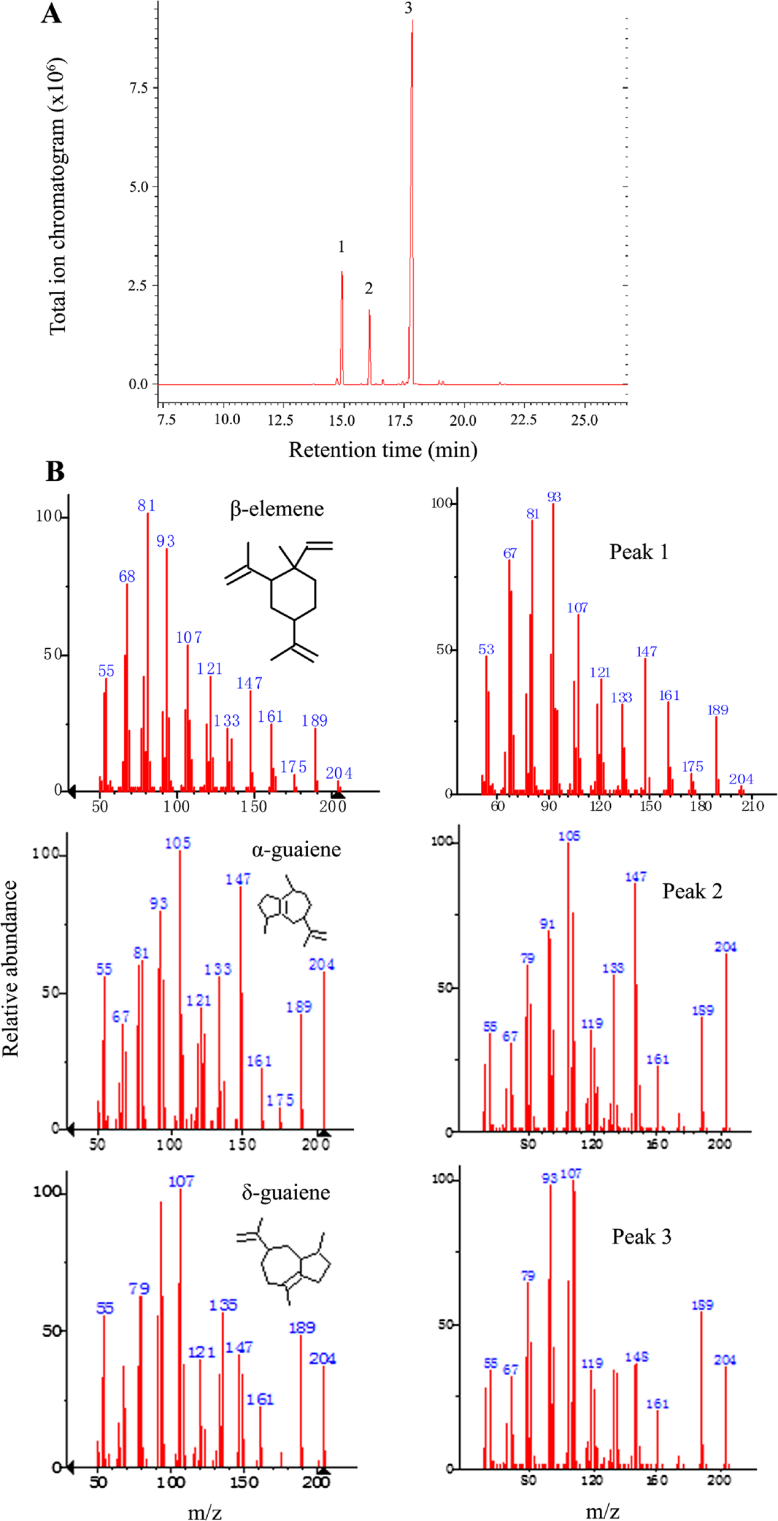
**Gas chromatography-mass spectroscopy profiles.** (**A**) Total ion chromatogram of the products formed by sesquiterpene synthases (ASSs) with farnesyl diphosphate as a substrate. (**B**) Mass spectra of the sesquiterpenes and their authentic standards. Peak 1 is β-elemene, peak 2 is α-guaiene, and peak 3 is δ-guaiene.

To confirm that *ASS1-3* encode active ASSs, we cloned them into a pET-28a vector and heterologously expressed them in *E. coli*. The fusion proteins were detected in soluble fractions (data not shown). The enzyme assays were performed using the geranyl diphosphate (C10), FPP (C15), and geranylgeranyl diphosphate (C20) substrates, and the reaction products were analyzed using GC-MS. The result showed that ASS1-3 did not accept geranyl diphosphate or geranylgeranyl diphosphate as a substrate, converting only FPP to terpene products. The 3 enzymes yielded the same compounds; the major product was identified as δ-guaiene (74.2%), and the minor products as β-elemene (16.3%) and α-guaiene (9.5%) (Figure 5).

### Expression of *ASSs* induced by MJ and identification of sesquiterpenes produced in MJ-treated calluses

**Figure 6:**
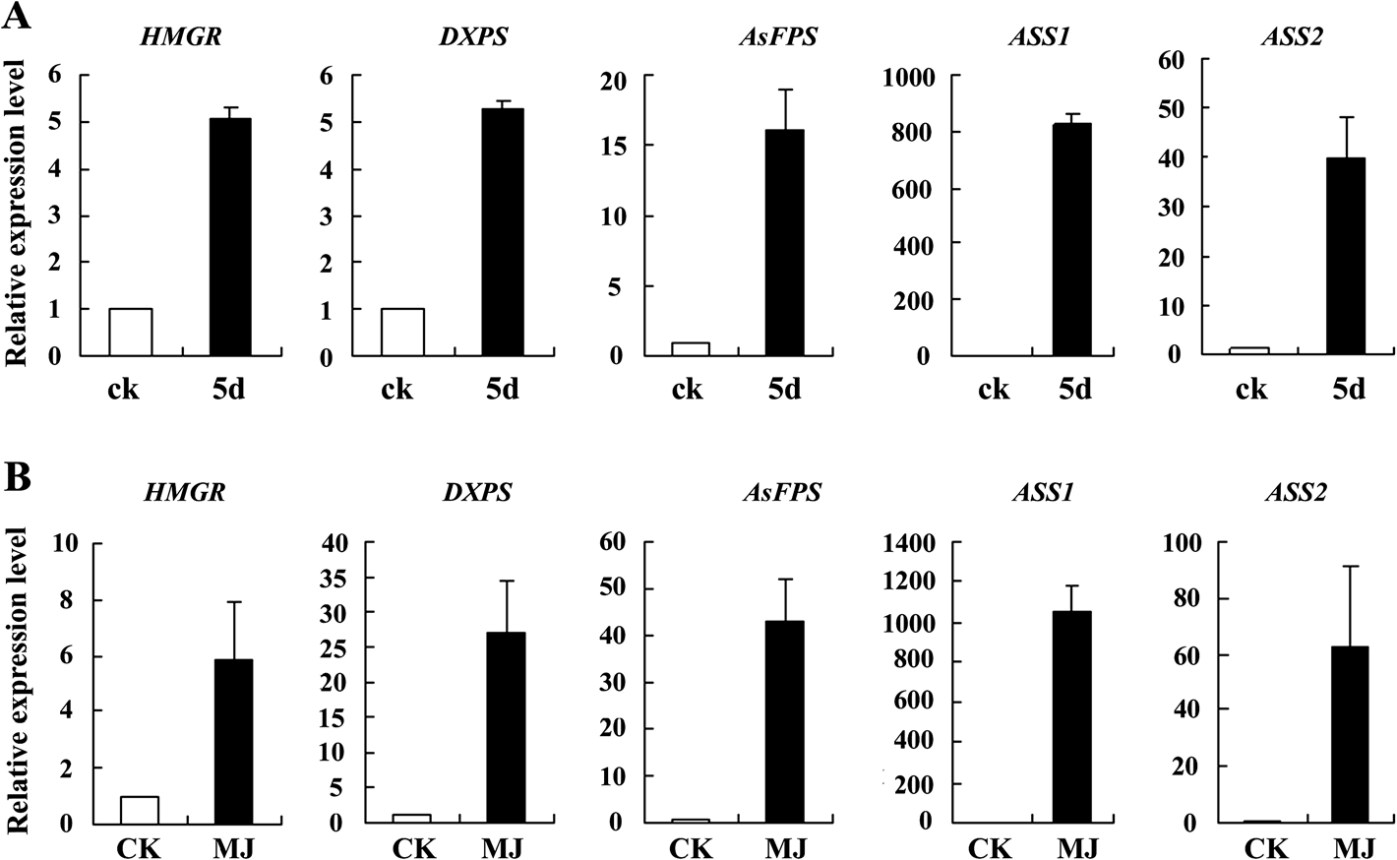
**Expression of 4 synthase genes in response to mechanical wounding and methyl jasmonate (MJ) treatment.** (**A**) In the stem of 3-year-old *A. sinensis* trees, mechanical wounding increased the mRNA levels of 4 genes that encode enzymes in the sesquiterpene biosynthesis pathway. Each value is the mean ± SE of 3 independent biological determinations. CK: healthy 3-year-old control trees; 5d: 3-year-old trees pruned after 5 days. (**B**) In *A. sinensis* calli, treatment with 100 μM MJ increased the mRNA levels of 4 genes that encode enzymes in the sesquiterpene biosynthesis pathway. *A*. *sinensis* calli were transferred to MJ-containing medium and cultivated for 5 days before being sampled for analysis. Each value is the mean ± SE of 3 independent biological determinations, and the data shown are relative ratios. The genes were named according to their functional annotation. CK: healthy calli controls; MJ: calli treated with MJ for 5 days.

We analyzed the expression profile of *ASS* using RT-PCR. Two pools of cDNAs, one derived from the wounded stems of the 3-year-old *A. sinensis* that comprised the 454 library and another from *A. sinensis* calluses treated with MJ, were used for the analysis. MJ is an essential signaling molecule that modulates plant secondary metabolism [24-26] and is capable of inducing the production of sesquiterpenes in *Aquilaria* spp. [5,27,28]. Therefore, MJ-treated calluses were also used to investigate the expression of *ASS*. The results demonstrated that *ASS1* and *ASS2* were both induced by either mechanical wounding or MJ treatment (Figure 6A, 6B). The expression of *ASS1* was upregulated significantly—approximately 800 times in response to mechanical wounding (see Figure 6A) and 1000 times in response to MJ treatment (see Figure 6B).

Furthermore, we tested the expression of other enzyme genes in sesquiterpene biosynthesis, including *HMGR*, *DXPS*, and *FPS*. They were also induced by mechanical wounding and MJ, but the upregulation rate was significantly weaker than that associated with *ASS1* and *ASS2*, fully demonstrating that *ASS1* is a typical stress-inducible gene. This result is consistent with those of solid-phase micro-extraction GC-MS analysis, showing that the production of sesquiterpenes increases in response to MJ treatment. In the untreated healthy callus samples, only 2 sesquiterpenes (δ-guaiene and α-guaiene) were detected, with total peak area of 11.47. In the MJ-treated callus samples, 4 sesquiterpenes were detected, with total peak area of 850.6—approximately 74 times higher (Table 3). Among the 4 sesquiterpenes, the content of δ-guaiene and α-guaiene was more than 88%, an increase of more than 67-fold relative to the healthy control. The results demonstrated that inducible expression of *ASS* is responsible for the formation of agarwood sesquiterpenes in *A. sinensis*.

**Table 3:** Kinds and relative contents of sesquiterpenes from the untreated healthy calli and the MJ-treated calli

**T**_**R**_	**Sesquiterpene materials**	**RI**	**Peaks areas (x10**^**8**^**)**	
			**Healthy**	**MJ-treated**
12.808	β- humulene	1212.378	-	7.94
13.008	α-guaiene	1217.037	5.52	248.6
13.483	α-humulene	1228.101	-	86.96
14.461	δ-guaiene	1250.883	5.95	507.1

T_R_: retention time.

RI: retention indices.

‘-’: not containing the substance.

### Candidate regulators response to wound signals

TFs are critical regulators of gene expression and environmental stress responses [26,29]. In our 454 unigene database (H + W), a total of 4,786 unigenes represented homologs of various TF families (Additional file 10: Table S3): 86 were annotated as belonging to the APETLA2 (AP2)/ethylene-responsive-element-binding family [30,31], 66 to the bZIP family [32], 108 to the MYB family [33-35], 150 to the MYB-related family, 169 to the bHLH family [35], and 99 to the WRKY family [36,37]. All of these TFs are reportedly stress-related [36-39], and some homologous genes have been identified as positive or negative regulators in the biosynthesis of secondary metabolites in other plants [11,40-42].

In defensive response and wound signal transduction, calcium signaling and mitogen-activated protein kinase (MAPK) cascades play important roles [25,43,44]. In our study, KEGG classification annotated 25 unigenes as being related to calcium signaling pathways and 41 to MAPK signaling pathways (Additional file 11: Table S4).

### RT-PCR analysis of candidate regulators response to wound signals

**Figure 7:**
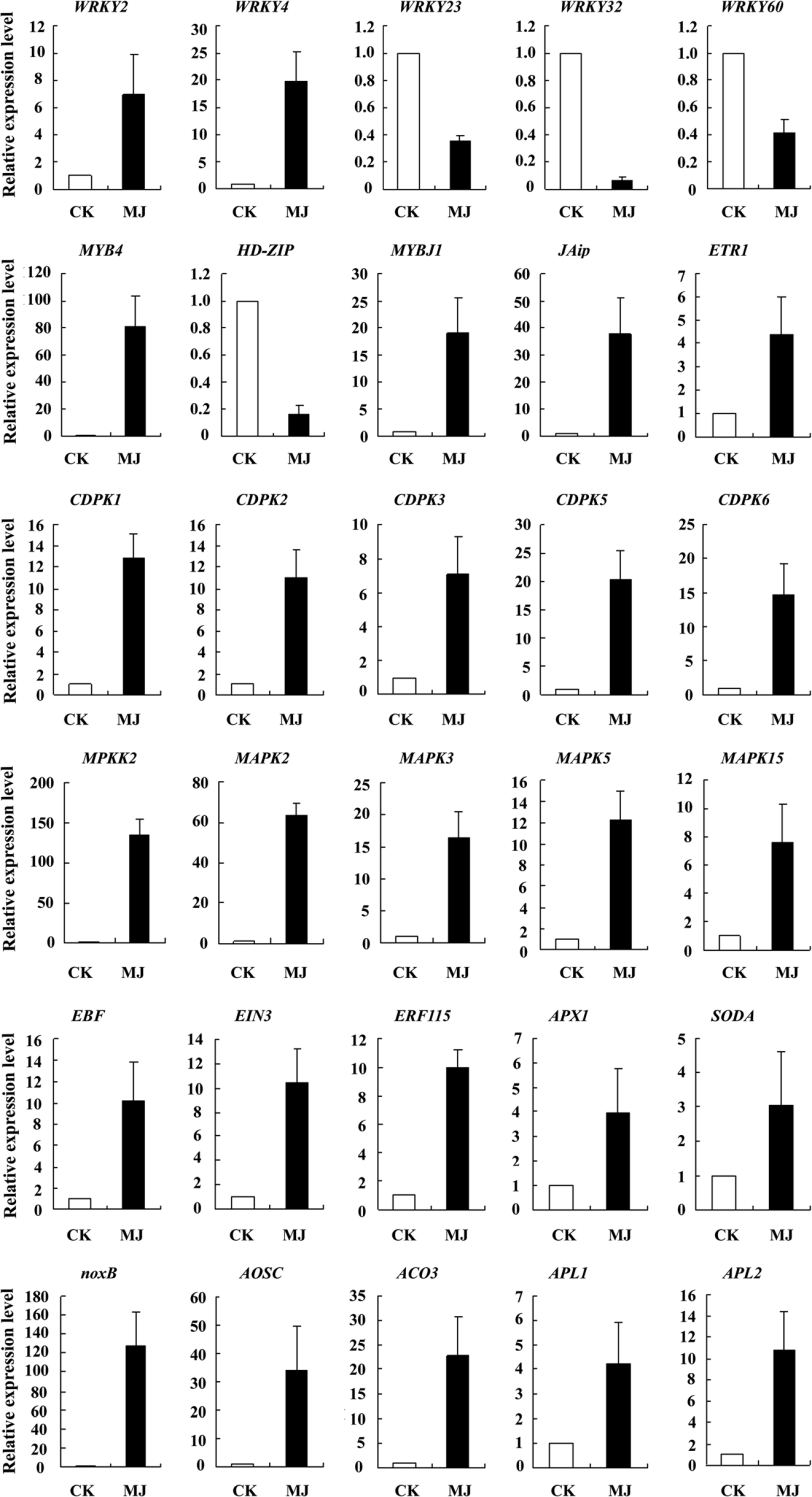
**Altered expression of a set of regulatory genes in response to MJ treatment.** Treatment with 100 μM MJ altered the mRNA levels of a set of regulatory genes that may be involved in wound signal transduction. Each value is the mean ± SE of 3 independent biological determinations, and the data shown are relative ratios. The genes were named according to their functional annotation. CK: healthy calli controls; MJ: calli treated with MJ for 5 days.

To confirm which of the candidate regulators mentioned above are related to wound signals, we performed RT-PCR analysis to test the effects of MJ on the expression of these genes. Overall, 30 expressed regulators were selected (Additional file 12: Table S5), including TFs *MYB* and *WRKY*, protein kinases *CDPK* and *MAPK*, and some regulators related to signal molecules MJ, ethylene, and hydrogen peroxide. As shown in Figure 7, the expression of most of these genes was elevated in response to MJ treatment, and only 4 TFs, *HD-ZIP*, *WRKY23*, *WRKY60*, and *WRKY32*, were downregulated by MJ. Among the upregulated genes, 17 were increased more than 10-fold. They were TFs *WRKY4* and *MYB4*, protein kinases *CDPK1*, *CDPK2*, *CDPK5*, *CDPK6*, *MPKK2*, *MAPK2*, *MPK3*, *MAPK5*, and JA-, ethylene-, or hydrogen peroxide-related regulators *JAip*, *RBF*, *EIN3*, *ERF115*, *noxB*, *AOSC*, *ACO3*. The expression of 5 annotated unigenes, *MPKK2*, *MAPK2*[45], *MYB4*[46], *WRKY4*, and *noxB*, the superoxide-generating NADPH oxidase [47,48], was coordinate with *ASS1*, showing elevation of 126.9-, 63.9-, 80.9-, 23.7-, and 95.2-fold, respectively. The expression profiles of these wound-signaling-related genes are essentially consistent with the idea that the expression of *ASS1* is induced by wounding but regulated through a complex underlying mechanism.

## Discussion

### *Aquilaria sinensis* transcriptome sequencing

Although *Aquilaria* is the most important plant source of agarwood and all *Aquilaria* spp. are listed in Appendix II of the Convention on International Trade in Endangered Species of Wild Fauna and Flora [49], little progress to date has been made in deciphering the mechanism of agarwood formation and genomic and genetic information is lacking. The NCBI EST database contained only a few hundred *Aquilaria* sequences before this study. The 454 pyrosequencing reported herein represents the first concrete effort to generate cDNA resources for *Aquilaria* spp. We obtained 89,137 unigenes from the H and W *A. sinensis* cDNA libraries (Table 1) and annotated them in detail at bioinformatics levels (see Additional file 3: Table S2, Additional file 4: Figure S2 and Additional file 5: Figure S3). We estimated the genome size of *A. sinensis* preliminarily at approximately 2 G using flow cytometry. In the absence of a haploid, genome sequencing is difficult to carry out with current sequencing technology. In the absence of complete genome sequences, transcriptome mining provides the sole option for the subsequent annotation of sequences. The data reported herein may be useful for further research on the functional genomics of *Aquilaria* spp.. Importantly, by comparing the unigenes in the W and H libraries, we obtained a large number of DEGs (see Figure 3). These data suggested that the transcriptome of *A. sinensis* changed significantly in response to mechanical damage and also provided resources for screening genes related to wound signaling and metabolism. KEGG annotations of these DEGs showed that most of the unigenes were related to metabolism processes, especially carbohydrate metabolism, and had higher abundance in the W library than in the H library (see Figure 4). The number of carbohydrate metabolism-related unigenes was slightly greater in the W library, indicating that starch and sugar metabolism in *A. sinensis* may be stimulated by wounding (see Figure 2).

The synthesis of sesquiterpenes starts with acetyl-coenzyme A (the MVA pathway) [8] or glyceraldehyde-3-phosphate and pyruvate (the DXP pathway) [10] from glycolysis. Therefore, agarwood formation may have some association with starch and sugar metabolism and is essentially consistent with our electron microscopy findings that a high number of starch granules exist in the healthy wood of *A. sinensis*, but when the wood is wounded, resin in the agarwood accumulates while the starch granules degrade (our unpublished data).

### *ASSs* are inducible enzymes that may be responsible for the formation of agarwood sesquiterpenes

Considering that sesquiterpenes are the most important components of agarwood [2,6,7], we tried to identify the genes that encode the enzymes involved in sesquiterpene biosynthesis, and 30 such unigenes were found in our database, including 4 categories of critical genes (*HMGR*, *DXPS*, *FPS*, and *ASS*) and other genes encoding synthases in sesquiterpene metabolism in *Aquilaria* spp. (Table 2). These data will be valuable for further studies of the terpene biosynthesis pathway. Using a homology-based PCR approach, we obtained 3 full-length cDNAs of *ASSs* (*ASS1-3*) with very similar amino acid sequences. Phenetic analysis showed that the evolutional distances among the 3 ASSs were quite small compared to those of sesquiterpene synthases from other plant species, except *A. crassna* (see Additional file 9: Figure S7). Heterologous expression in *E. coli* and enzyme assays showed that ASS1-3 had ASS activity, and the major product was revealed to be δ-guaiene (see Figure 5). Expressions analysis with real-time PCR showed that *ASS1* was barely detectable in either the healthy tissues or the calluses of *A. sinensis*, whereas in the wounded tissues or MJ-treated calluses, *ASS1* expression was increased almost 1000-fold (see Figure 6). Furthermore, GC-MS analysis showed that the major product was δ-guaiene, essentially consistent with its enzyme reaction, and the sesquiterpene content was increased 74-fold (Table 3), clearly demonstrating that *ASS1* expression was wound-induced and is responsible for the formation of agarwood sesquiterpenes in *A. sinensis*.

Previous reports have shown that MJ can induced 3 sesquiterpenes (α-guaiene, α-humulene, and δ-guaiene) in cultured cells of *A. crassna*[5,27,28] and be produced by the enzymatic reaction of cloned enzymes. Of the 5 clones (AcC1, AcC2, AcC3, AcC4, and AcL154), AcC1 and AcL154 have no TPS activity that might indicate pseudogenes [5]. The 3 clones (*ASS1-3*) in this study have high homology with AcC4 and AcC3 (see Additional file 9: Figure S7) and catalyzed the same major product. Thus, the present experiments allow us to identify ASS1-3 as active enzymes of *A. sinensis*, and their inducible expression is responsible for the formation of agarwood sesquiterpenes.

### Wounding induces *ASSs* expression via a complex signaling pathway

Research has shown that only after wounding (including mechanical wounding, chemical induction, and fungal infection, among others) can agarwood be induced in the healthy wood of *Aquilaria* spp*.*[6,14-17]. It is, therefore, reasonable to suppose that the early period of agarwood formation is a process of wound signaling transduction before the strong expression of ASSs. Previous studies in other plant species have shown that TFs play important roles in signal transduction and gene expression. Some TFs, such as WRKY, MYB, MYC, bZIP, ERF, and EIN3 are involved in plant defense and stress responses [34,36,39]. Our 454 unigene database contained 4,786 putative TFs. Some homologous genes of these TFs, such as *AP2*, *WRKY*, and *MYC*, have been identified as positive regulators of sesquiterpene synthases in other plant species [11-13,42]. In *Catharanthus roseus*, the TF ORCA3 regulates the biosynthesis of terpenoid indole alkaloids and plays an important role in JA response [42]; in cotton, the TF GaWRKY1 positively regulates the expression of the sesquiterpene synthase (+)-δ-cadinene synthase gene and the synthesis of gossypol sesquiterpene [11]. Similarly, in *A. annua*, AaWRKY1 and 2 JA-responsive AP2/ethylene response factor (ERF) TFs, ERF1 and ERF2, are involved in regulating the expression of the sesquiterpene synthase ADS and the production of artemisinin [12,49]; in *Arabidopsis*, MYC2 interacts with DELLA proteins and positively regulates the expression of TPS21 and TPS11 [13].

Reversible protein phosphorylation has been proven to be an important regulatory mechanism in the wound signaling pathway, and studies on *Arabidopsis*, tomato, and tobacco have indicated that the MAPK cascade is a widespread pathway [44]. In addition, calcium and calcium-binding proteins may play some roles in the regulation of early wound responses by elevating intracellular calcium levels and changing the phosphorylation patterns of proteins [50]. In this study, 41 unigenes were annotated as related to the MAPK pathway and 25 to calcium signaling, which may play roles in the wound response of agarwood formation.

In the present experiment, 30 DEGs related to wound signal were selected to investigate whether they are co-expression with *ASS1* in response to MJ. The results showed that 26 were upregulated and 4 were downregulated. Of these, 5 unigenes, including the TFs *MYB4* and *WRKY4*, the protein kinases *MAPK2* and *MPKK2*, and the NADPH oxidase *noxB*, were significantly upregulated by MJ. The downregulated genes were *WRKY23*, *WRKY32*, *WRKY60*, and *HD-ZIP* (see Figure 7). The *ASS1* promoter has been cloned and scanned with PLACE (http://www.dna.affrc.go.jp/htdocs/PLACE/signalscan.html). It contains some *cis*-acting elements that could be combined specifically by certain TFs, including WRKY, MYB, and MYC (data to be published elsewhere). Therefore, we speculate that TFs *MYB4* and *WRKY4* may positively regulate the expression of sesquiterpene synthase, and the downregulated genes may negatively regulate its expression. The generation of hydrogen peroxide has been confirmed to occur both locally and systematically, and its main source is the plasma-membrane-bound NADPH oxidase. In the present study, NADPH oxidase noxB was significantly upregulated by MJ, which is consistent with the theory that MJ induces the production of hydrogen peroxide [51-54]. MAPK signal transduction pathways are widespread mechanisms in eukaryotic cells that couple environmental responses and transcriptional regulation [55,56]. Two coupled components of the MAPK cascade, MAPK2 and MAPKK2, were significantly upregulated by MJ, demonstrating they may be involved in MJ-mediated signaling. Calcium is a prevalent messenger molecule in higher plant cells that can mediate plant responses to external signals and regulate a variety of physiological processes. In our experiments, 4 CDPKs (*CDPK1*, *CDPK2*, *CDPK5*, *CDPK6*) were upregulated more than 10-fold (see Figure 7).

**Figure 8:**
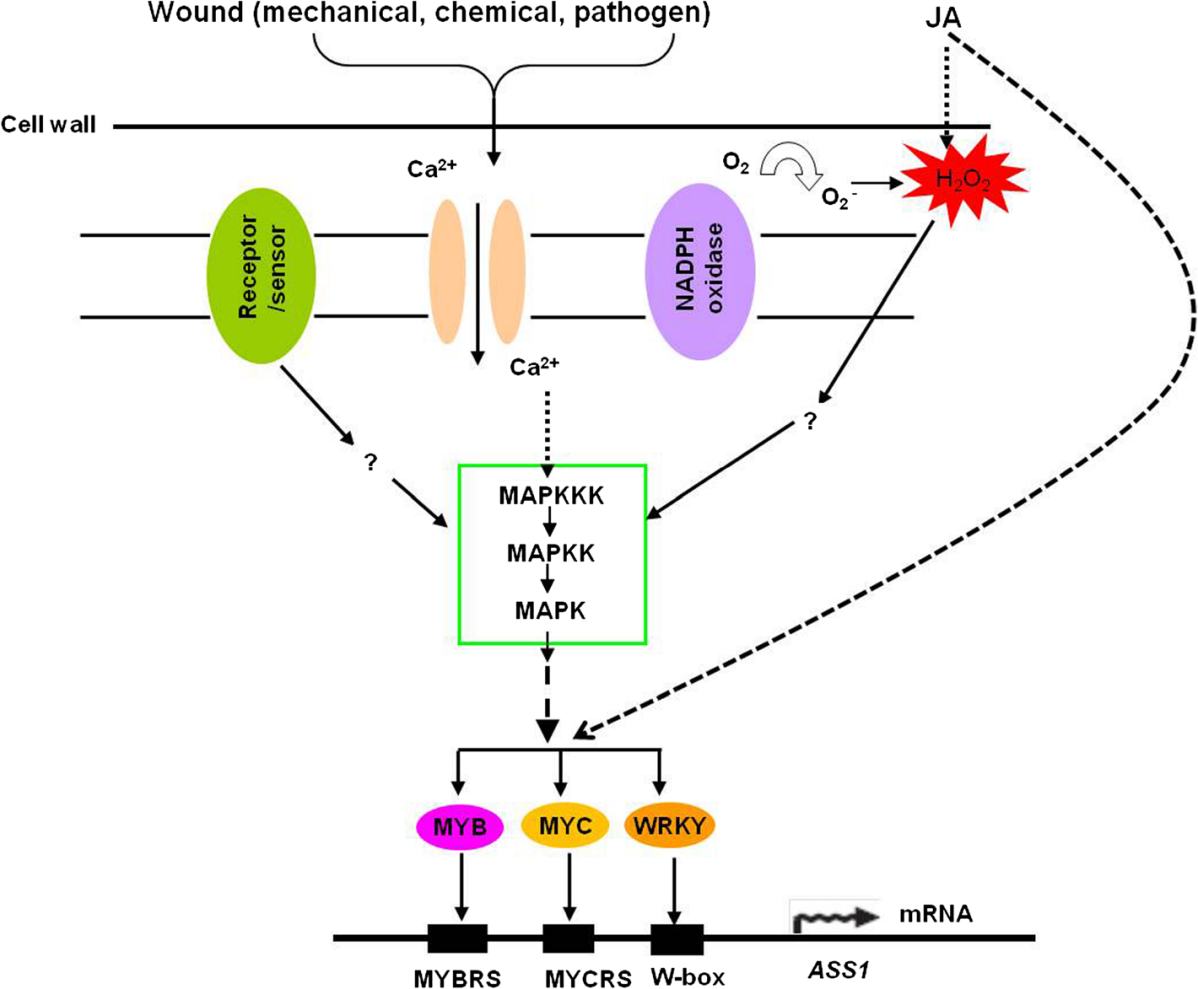
**Proposed mechanism of wound-induced sesquiterpene biosynthesis and regulation.** Wound signals stimulate the mitogen-activated protein kinase (MAPK) cascade and phosphorylate transcription factors downstream. Jasmonic acid (JA) signals occur via the hydrogen peroxide pathway or directly activate the transcription factors downstream. The activated transcription factor binds to the *cis*-acting element in the promoter of *ASS1* and starts the transcription of sesquiterpene synthase. Dashed line indicates a possible pathway; ‘?’ indicates an unknown factor. MYBRS, MYB recognized sequence; MYCRS, MYC recognized sequence.

Based on these results, we proposed a model to explain the mechanism by which *ASS1* is modulated at the transcriptional level (Figure 8). In this model, the MAPK cascade is activated by wound signals and phosphorylates downstream TFs such as MYB or WRKY. JA signaling may activate the TFs downstream via the hydrogen peroxide or JA-JAZ-TF pathways [57]. Activated TFs bind to the *cis*-acting element in the promoter of *ASS1* and initiate *ASS1* transcription. *ASS2* is also inducible and may be influenced by the same regulatory mechanisms as those affecting *ASS1*. The results reported herein enhance understanding of wound-induced sesquiterpene synthases expression and agarwood formation. Future studies will focus on verifying this proposed model based on the promoter of *ASS1* that we have cloned. We will identify which TFs interact with the *ASS1* promoter, confirm whether the MAPK cascade is involved in the wound signaling pathway, and determine the roles of them in ASS1 expression. Clarifying the wound-induced sesquiterpene biosynthesis pathway will help to develop strategies for controlling agarwood production and for the long-term protection of *Aquilaria* spp..

## Conclusions

A comprehensive transcriptome analysis was conducted for healthy and wounded stems of *A. sinensis* in this study. A total of 89,137 unigenes with an average length of 678.65 bp were obtained, and they were annotated in detail at the bioinformatics levels. These data should be useful for further research on the functional genomics of *A. sinensis* and other *Aquilaria* spp.. Transcripts related to sesquiterpene metabolism and regulation were highlighted. Three full-length cDNAs of sesquiterpene synthase (*ASS1-3*) were cloned and identified. Through MJ-inducible expression pattern experiments and GC-MS analysis, we found that their expression was significantly induced by MJ, and sesquiterpenes content was elevated accordingly. The expression of a number of important regulatory genes was detected using RT-PCR and indicated that some of them may participate in one or more processes of wound signal transduction, transcriptional regulation of *ASS*, and biosynthesis of agarwood sesquiterpenes. Based on the analysis of *ASS1* promoter, we confirmed preliminarily that TFs *MYB4* and *WRKY4* may be key positive regulators of *ASS1* expression. These results provide a better understanding of sesquiterpene biosynthesis and regulation in *Aquilaria* spp*.*, and provide valuable clues and a basis for elucidating the molecular mechanism of agarwood formation.

## Methods

### Plant materials for cDNA library construction

Three-year-old *A. sinensis* trees were planted in Hainan Province of China. The stems were pruned with a knife, and samples were taken from 6 cm below the pruned site at various times after pruning: 2 h, 4 h, 6 h, 8 h, 12 h, 24 h, 3 d, and 5 d. All of the stems from the wounded plants were mixed in equal proportion to generate material for the wounded library, and the stems cut from healthy trees were used to generate material for the healthy library.

### Plant materials for real-time PCR (RT-PCR) and gas chromatography–mass spectrometry (GC-MS) analysis

Calluses originating from the stems of the healthy *A. sinensis* plants were used to investigate the effect of MJ on candidate genes expression and sesquiterpenes production. Calluses were transferred to 100 μM MJ-containing medium and allowed to grow for 5 days before being sampled for analysis.

### Library construction and 454 sequencing

Total RNA was isolated from the wounded and healthy materials in the libraries using a TRIzol kit (Invitrogen, USA) and purified to enrich messenger RNA (mRNA) and exclude transfer RNA (tRNA) and ribosomal RNA (rRNA) using an mRNA purification kit (Promega, USA) according to manufacturer instructions. The mRNA was reverse-transcribed with PowerScript II (Takara, Japan) using SMART IV oligonucleotide PCR primers (5^′^-AAGCAGTGGTATCAACGCAGAGTGGCCATTACGGCCGGG-3^′^) and a CDS III/3^′^ PCR primer (5^′^-ATTCTAGAGGCCGAGCGGCCGACATG -d[T]30 N-1 N-3^′^). Long-distance PCR for double-stranded cDNA amplification was performed with LA Taq enzyme (Takara, Japan) for 25 cycles (95°C for 30 s, 68°C for 8 min) according to the SMART cDNA Library Construction Kit user manual. Finally, the double-stranded cDNA was purified using a DNA purification kit (Qiagen, Germany) to generate high-quality cDNA. Approximately 10 μg cDNA was sheared and used for 454 sequencing. The cDNA sample was end-repaired and ligated to adapters. Streptavidin bead enrichment, DNA denaturation, and emulsion PCR were performed according to a procedure described by Margulies [58]. Each portion of the preparations from the healthy and wounded libraries were loaded on a full plate and sequenced in one run.

### 454 data processing and assembly

A Perl script was used to remove vector sequences and Poly(A)/(T) tails from the original sequences. Reads shorter than 100 bp were deemed low quality reads and removed before assembly. The remaining high-quality reads were assembled using MIRA [59] to construct unique consensus unigenes, with the parameter --job = de novo, est, normal, 454 -SK: mnr = yes -SK: rt = 2454_SETTINGS -LR: mxti = no, and then to remove the redundancy of unigenes, UIcluster [60] (version 2.0) was used to cluster the unigenes, with the parameter --tryRevC -R 50 –M 40 –E 2, finally the clusters of unigenes were assembled by Cap3 [61], with parameter –o 100 –p 95.

### Functional annotation and classification

#### NT and NR

The unigenes were searched against the NCBI’s (http://www.ncbi.nlm.nih.gov) Non-redundant nucleotide database (NT) using the BLASTN program and against the Non-redundant protein database (NR) using the BLASTX program. For both searches, an *E* value ≤ 1e-5 was used.

#### SWISS-PROT

The unigenes were searched against the SWISS-PROT database (http://www.expasy.ch/sprot) using the BLASTX program with an *E* value ≤ 1e-10.

#### KEGG and KEGG Orthology

Unigenes were compared with the Kyoto Encyclopedia of Genes and Genomes database (KEGG, release 50; http://www.genome.jp/kegg/) [62] using the BLASTX program with an *E* value ≤ 1e-10. A Perl script was used to retrieve KEGG Orthology (KO) information from the BLAST results and then to establish pathway associations between the unigenes and KEGG.

#### Interpro and Gene Ontology

InterProScan [63] release 16.0 was used to annotate the InterPro domains of the unigenes, and then the functional assignments were mapped onto the Gene Ontology (GO) [64]. The WEGO tool [65] was used to assign GO classifications and draw GO trees.

#### Differentially expressed unigenes detection

The IDEG6 web tool [19] was used to identify unigenes that showed statistically significant differences in relative abundance (as reflected by the total count of individual sequence reads) between the two libraries. Comparisons between the wounded and healthy samples were performed using the general Chi2’ method, which has been proven to be the most efficient test. The unigenes with *P* ≤ 0.01 were deemed to be significantly different between the two samples. This is similar to the credibility intervals approach that was employed earlier for the analysis of SAGE data [66].

#### Transcription Factors annotation

Assembled transcripts were searched against all the transcription factor protein sequences of Plant transcription factor database [67] (PlnTFDB, version 3.0) using BLASTX with an E-value cut-off of ≤ 1e − 10, and positives identity ≥50%.

### Phenetic analysis

The translated amino acid sequences and the putative amino acid sequences derived from the NCBI database search were aligned using the CLASTAL W program. Evolutionary distances were calculated using the Poisson correction method, and a neighbor-joining (NJ) tree was constructed with MEGA4 [68]. Bootstrap values were obtained after 1,000 replications.

### Quantitative real-time PCR (RT-PCR) analysis

To investigate the expression of the mRNA of candidate genes, RT-PCR was performed using the Bio-Rad IQ-5 Real-Time System essentially according to the manufacturer’s instructions. Total RNA was isolated from three-year-old *A. sinensis* stems and calluses using a Total RNA Purification Kit (LC Science, USA) with an on-column DNA digestion according to the manufacturer’s instructions. The RNA samples were then reverse transcribed using the Superscript II RT kit (Invitrogen, USA). The amplification of the *AcHistone* gene [5] was used as an internal control. The efficiency of annealing of the oligonucleotide PCR primer sequences (listed in Additional file 13: Table S6) was evaluated using the Primer 5.0 program. The cDNA was amplified in a 10 μL volume using SYBR Premix Ex Taq (TaKaRa, Japan) and a DNA Engine Opticon 2 thermal cycler with the following program: one cycle of 95°C for 10 s and 40 cycles of 94°C for 10 s and 60°C for 20 s. The amplification of the target genes was monitored every cycle by SYBR-green fluorescence. The Ct (threshold cycle), defined as the PCR cycle at which a statistically significant increase of reporter fluorescence was first detected, was used as a measure for the starting copy numbers of the target gene. Relative quantization of the target gene expression level was performed using the comparative Ct method. Three technical replicates were performed for each experiment. For all the RT-PCR analyses, the assays were repeated three times along with at least three independent repetitions of the biological experiments, and the means of the three biological experiments were calculated to estimate gene expression.

### GC-MS analysis and identification of sesquiterpene components in *A. sinensis* calluses

GC-MS analysis was performed with a Varian 450 GC (USA) equipped with a VF-5MS capillary column (30 m × 0.25 mm internal diameter; film thickness, 0.25 μm), and a Varian 300 mass spectrometer with an ion-trap detector in full-scan mode under election impact ionization (70 eV). The carrier gas was helium, and the flow rate was 1 mL/min. The injections were performed in splitless mode at 250°C. The healthy and MJ-treated calluses were powdered and took the same weight (0.5 g) to place into the 15 mL sample bottle, and balanced for 30 min in 60°C water. The fused silica fiber of the Solid Phase Micro Extraction (SPME) was introduced into the headspace above the sample, adsorbing the volatile components for 30 min. The adsorbed components were followed by a thermal desorption process by introducing the SPME fiber into the injection port of a gas chromatography. The program was immediately started, and the fiber was removed after 10 min. The condition for GC-MS and identification of sesquiterpenes compounds were performed according to previously described procedures [6]. The two fibers were injected separately and ran in the same program, and the relative components were obtained by peak areas normalization without applying correction factors [6]. We didn’t use the internal standard, for it is difficult to choose one suiting for both of the samples. Peaks areas can reflex the content of concerning compound in the two different samples in some extent, as the weight of the two samples and the analytical methods were identical.

### Heterologous expression of *ASS* genes in *Escherichia coli* and enzyme assays

The full-length sequence of *ASS1* was cloned into the XhoI (5^′^ end)/BamHI (3^′^ end) sites of a pET-28a vector (Novagen) and expressed in *E. coli* as a His-tag fusion protein using the following primers: forward primer, 5^′^-CCGCTCGAGATGTCTTCGGCAAAACTAGGT-3^′^ and reverse primer, 5^′^-CGGGATCCGATTTCAATAGCATGACGCAAC-3^′^ (XhoI and BamHI sites are indicated with underlining). To ensure that no errors were introduced by the PCR, the construct was checked with sequencing. Fusion proteins in the *E. coli* BL21 (DE3) cells were induced through treatment with 1 mM isopropylthio-β-galactoside (IPTG) for 12 h at 30°C and 200 rpm. The protein purification was performed using a Profinia Protein Purification System (Bio-Rad, USA). Bacterial lysates and purified enzyme samples were examined on SDS-PAGE gels to visualize expected proteins. Protein concentrations were determined with the Bradford assay using bovine serum albumin as the standard. The enzyme reaction was performed according to the method described by Kumeta & Ito (2010), with minor modification. The reaction was performed in a 4-mL vial with a solid-top polypropylene cap using 50 mL of crude protein extract in a final volume of 200 mL containing Tris–HCl buffer (25 mM, pH 7.0) supplemented with 10% glycerol, 100 mM MgSO4, 5 mM DTT, and 46 mM FPP. After incubation at 30°C for 4 h, a SPME fiber was inserted into the headspace of the vial to collect volatiles for 30 min and then transferred to the injection port of a GC-MS system for desorption. The GC-MS system was described above and the operating parameters were essentially according to Kumeta & Ito [5]. The identification of sesquiterpenes was based on a comparison of retention times and mass spectra with authentic standards.

## Competing interests

The authors declare that they have no competing interests.

## Authors’ contributions

YHX participated in the design of the study, analyzed the data, performed the real-time PCR experiment and drafted the manuscript. ZZ contributed to the sample collection, participated in the design of the study and analysis of the data. MXW performed the cloning and identification of three sesquiterpene synthase genes. JHW initiated the project, helped conceive the study, and revised the manuscript. HJC participated in the GC-MS analysis. ZHG, HML, XLZ and WLL participated in the discussion of the result and helped to revise the manuscript. CS helped to analysis the result and with the phenetic analysis. YY and HM helped to the samples collection. All authors read and approved the final manuscript.

## Supplementary Material

Additional file 1: Figure S1 Sesquiterpene biosynthesis pathway in plants. Click here for file

Additional file 2: Table S1 Distribution of the number of reads in the *Aquilaria sinensis* unigenes. Click here for file

Additional file 3: Table S2 Summary of annotation of the *A. sinensis* 454 assembled unigenes. Click here for file

Additional file 4: Figure S2 Comparison of healthy and wounded tissue libraries based on GO terms. Click here for file

Additional file 5: Figure S3 Functional classification of differentially expressed genes based on GO terms. Click here for file

Additional file 6: Figure S4 Phenetic analysis of the proteins translated from the full-length AsFPS sequence from *Aquilaria sinensis* and some characterized farnesyl diphosphate synthase sequences from other plant species. Bootstrap values after 1000 replications are shown on the branches. Click here for file

Additional file 7: Figure S5 Alignment of nucleotide sequences for 3 sesquiterpene synthase (*ASS*) genes. Click here for file

Additional file 8: Figure S6 Alignment of deduced amino acid sequences for 3 ASSs. Click here for file

Additional file 9: Figure S7 Phenetic analysis of 3 full-length sesquiterpene synthase sequences from *A. sinensis* (ASS1-3) and some characterized sesquiterpene synthase sequences from other plant species. Bootstrap values after 1000 replications are shown on the branches. Click here for file

Additional file 10: Table S3 Major transcription factors identified from *A. sinensis*. Click here for file

Additional file 11: Table S4 Unigenes annotated as being related to the calcium signaling and mitogen-activated protein kinase signaling pathways. Click here for file

Additional file 12: Table S5 Candidate regulators used for RT-PCR analysis. Click here for file

Additional file 13: Table S6 Primers used in this study. Click here for file
