# Supplementary material for: Identification of genes related to agarwood formation: transcriptome analysis of healthy and wounded tissues of Aquilaria sinensis
Source: BMC Genomics. 2013 Apr 8;14:227. doi: 10.1186/1471-2164-14-227 (PMC3635961; doi:10.1186/1471-2164-14-227)

**Additional file 1: Figure S1. Sesquiterpene biosynthesis pathway in plants.** HMGS, HMG-coenzyme A (CoA) synthase; HMG-CoA, 3-hydroxy-3-methylglutaryl CoA; HMGR, HMG-CoA reductase; MVA, mevalonic acid; MK, MVA kinase; MVAP, mevalonic acid 5-phosphate; PMK, MVAP kinase; MVAPP, mevalonic acid 5-diphosphate; MPD, MVAPP decarboxylase. GA-3-P, glyceraldehyde 3-phosphate; DOXP, 1-deoxy-d-xylulose-5-phosphate; DXPS, DOXP synthase; DXR, DOXP reductoisomerase; MEP, 2-C-methyl-d-erythritol 4-phosphate; CMS, CDP-ME synthase; CDP-ME, 4-diphosphocytidyl-2-C-methyl -d-erythritol; CMK, CDP-ME kinase; CDP-ME2P, 4-diphosphocytidyl-2-C-methyl-d-erythritol 2-phosphate; MCS, ME-2,4cPP synthase; ME-2,4cPP, 2-C-methyl-d-erythritol 2,4-cyclodiphosphate; HDS, HMBPP synthase; HDR, HMBPP reductase. HMBPP, 1-hydroxy-2-methyl-2-(E)-butenyl 4-diphosphate; IPP, isopentenyl diphosphate; DMAPP, dimethylallyl diphosphate; FPP, farnesyl diphosphate; FPS, farnesyl diphosphate synthase.


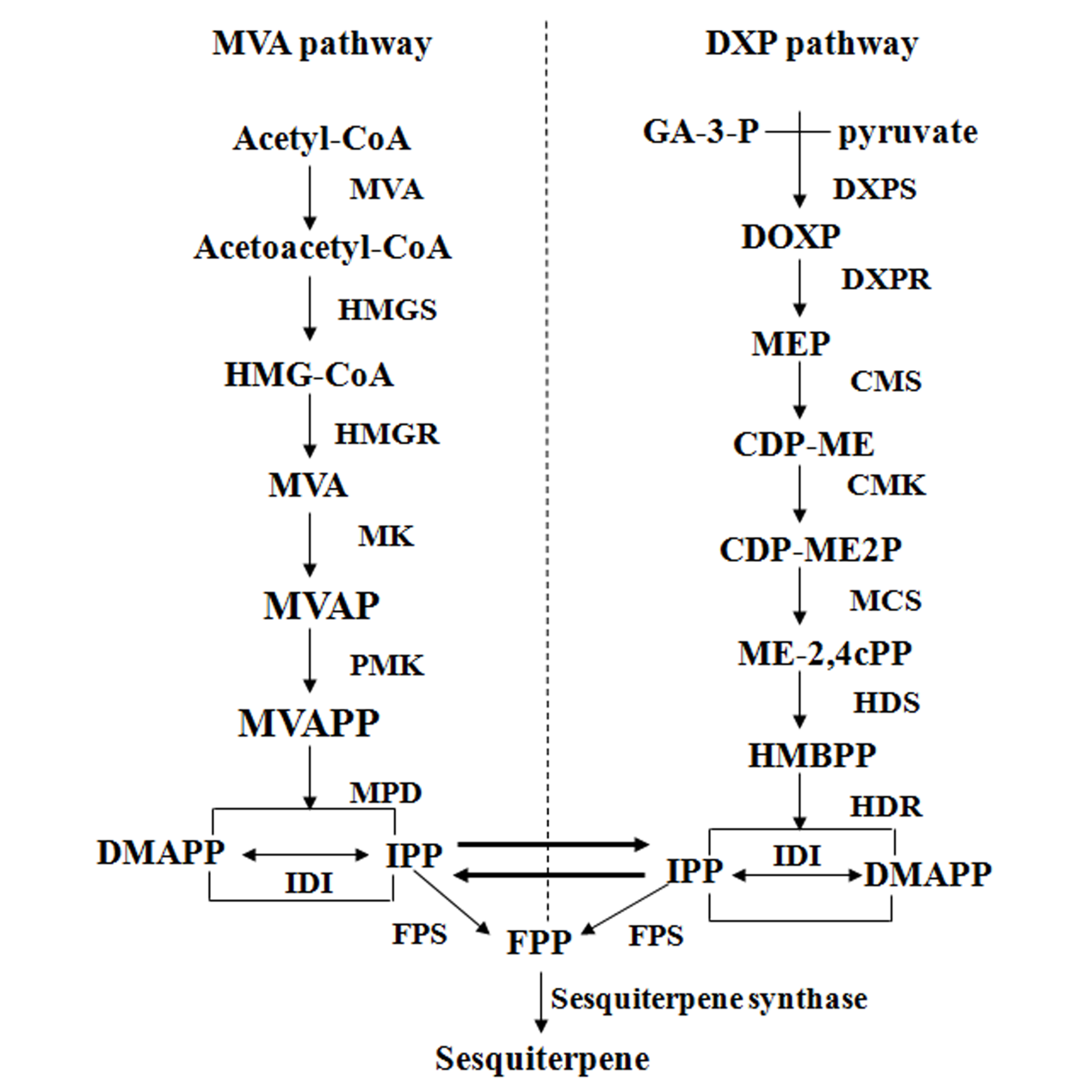

Supplement: Additional file 1: Figure S1 — Sesquiterpene biosynthesis pathway in plants. [file 1471-2164-14-227-S1.docx]
