# Supplementary material for: Identification of genes related to agarwood formation: transcriptome analysis of healthy and wounded tissues of Aquilaria sinensis
Source: BMC Genomics. 2013 Apr 8;14:227. doi: 10.1186/1471-2164-14-227 (PMC3635961; doi:10.1186/1471-2164-14-227)
Supplement: Additional file 2: Table S1 — Distribution of the number of reads in the Aquilaria sinensis unigenes. [file 1471-2164-14-227-S2.docx]

**Additional file 2: Table. S1. Distribution of number of reads in *A. sinensis* unigenes**

| **No. reads members** | **No. unigenes** | **Percent of unigenes（%）** |
| --- | --- | --- |
| 1 | 4247 | 4.8 |
| 2 | 28658 | 32.2 |
| 3 | 14145 | 15.9 |
| 4-5 | 14184 | 15.9 |
| 6-10 | 12744 | 14.3 |
| 11-20 | 7255 | 8.1 |
| 21-50 | 5093 | 5.7 |
| 51-100 | 1787 | 2.0 |
| >100 | 1024 | 1.1 |
| Max Unigenes Size is 3002 | | |

Unigenes Size: the number of reads in Unigenes.
