# Supplementary material for: Identification of genes related to agarwood formation: transcriptome analysis of healthy and wounded tissues of Aquilaria sinensis
Source: BMC Genomics. 2013 Apr 8;14:227. doi: 10.1186/1471-2164-14-227 (PMC3635961; doi:10.1186/1471-2164-14-227)
Supplement: Additional file 3: Table S2 — Summary of annotation of the A. sinensis 454 assembled unigenes. [file 1471-2164-14-227-S3.docx]

**Additional file 3: Table S2. Annotation summary of the *A. sinensis* 454 assembled unique sequences.**

| **Annotation Database** | **Annotation Number** | **Annotation Percent** | **E cutoff** | **Database version** |
| --- | --- | --- | --- | --- |
| Nt | 22051 | 24.7 | 1e-5 | 20100810 |
| Nr | 38159 | 42.8 | 1e-5 | 20100810 |
| Swissprot | 15280 | 17.1 | 1e-10 | 20100808 |
| Kegg | 29389 | 33.0 | 1e-10 | Release55 |
| COG | 7591 | 8.5 | 1e-10 | No Version |
| Interpro | 19780 | 22.2 | Interproscan 4.4 | Release27.0 |
| GO | 14609 | 16.4 |  |  |
