# Supplementary material for: Identification of genes related to agarwood formation: transcriptome analysis of healthy and wounded tissues of Aquilaria sinensis
Source: BMC Genomics. 2013 Apr 8;14:227. doi: 10.1186/1471-2164-14-227 (PMC3635961; doi:10.1186/1471-2164-14-227)

**Additional file 4: Figure S2.** Comparison of healthy and wounded tissue libraries based on GO terms. The results are summarized in three main categories: biological process, cellular component and molecular function. The right y-axis indicates the number of genes in a category. The left y-axis indicates the percentage of a specific category of genes in that main category.


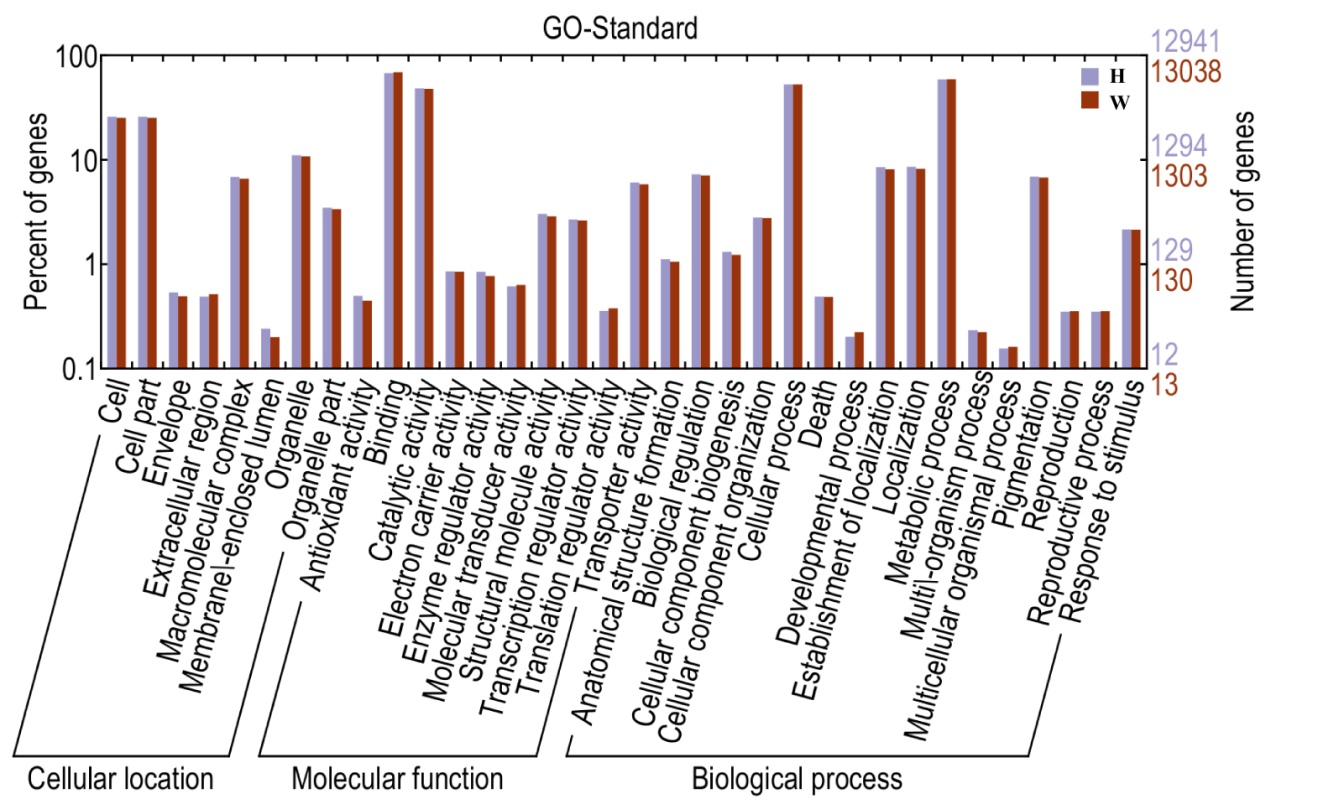

Supplement: Additional file 4: Figure S2 — Comparison of healthy and wounded tissue libraries based on GO terms. [file 1471-2164-14-227-S4.docx]
