# Supplementary material for: Identification of genes related to agarwood formation: transcriptome analysis of healthy and wounded tissues of Aquilaria sinensis
Source: BMC Genomics. 2013 Apr 8;14:227. doi: 10.1186/1471-2164-14-227 (PMC3635961; doi:10.1186/1471-2164-14-227)

**Additional file 6: Figure S4.** Phenetic analysis of the proteins translated from the full-length *AsFPS* sequence from *Aquilaria sinensis* and some characterized farnesyl diphosphate synthase sequences from other plant species. Bootstrap values after 1000 replications are shown on the branches.


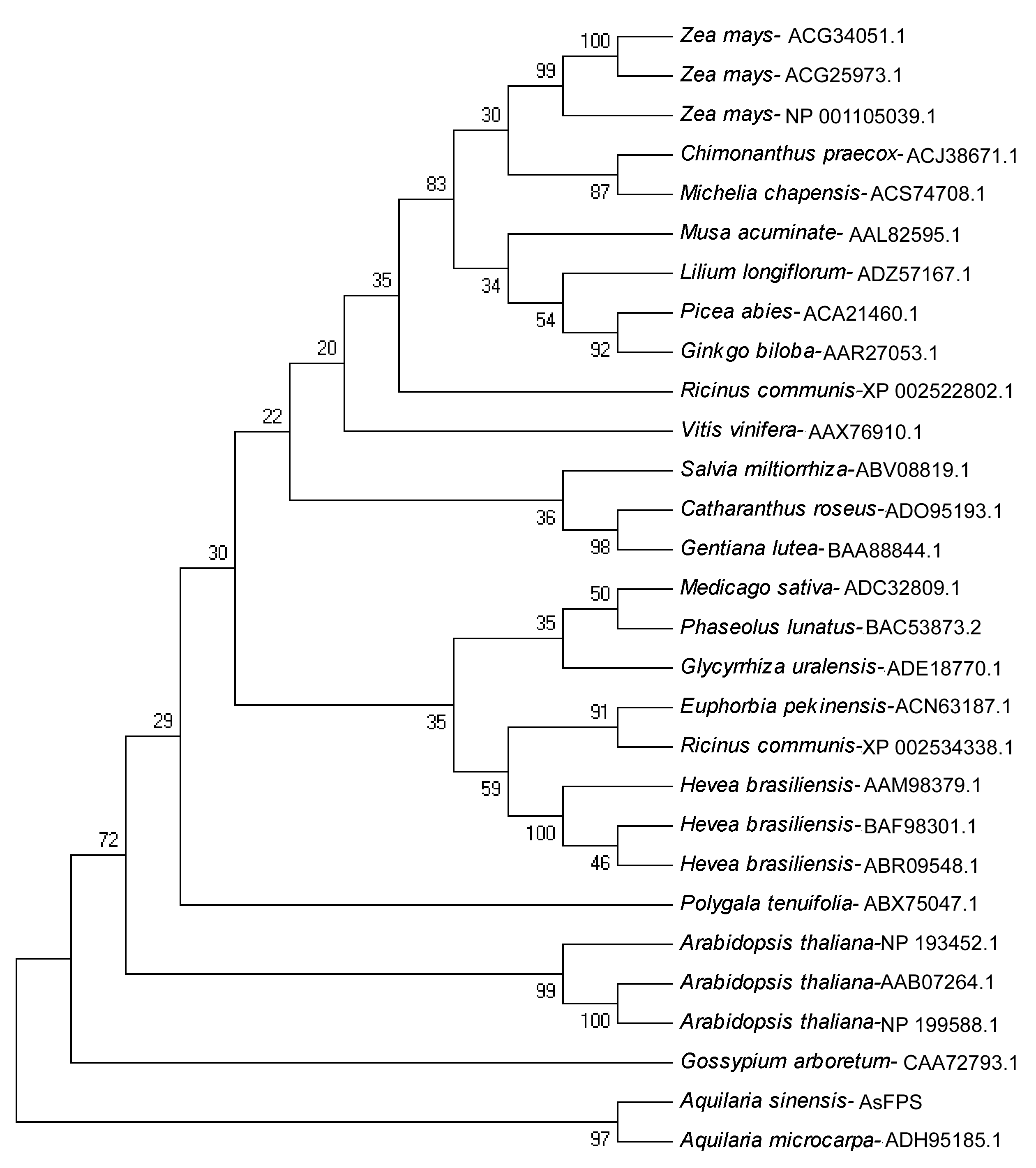

Supplement: Additional file 6: Figure S4 — Phenetic analysis of the proteins translated from the full-length AsFPS sequence from Aquilaria sinensis and some characterized farnesyl diphosphate synthase sequences from other plant species. Bootstrap values after 1000 replications are shown on the branches. [file 1471-2164-14-227-S6.docx]
