# Supplementary material for: Identification of genes related to agarwood formation: transcriptome analysis of healthy and wounded tissues of Aquilaria sinensis
Source: BMC Genomics. 2013 Apr 8;14:227. doi: 10.1186/1471-2164-14-227 (PMC3635961; doi:10.1186/1471-2164-14-227)

**Additional file 7: Figure S5.** Alignment of nucleotide sequences for 3 sesquiterpene synthase (*ASS*) genes.


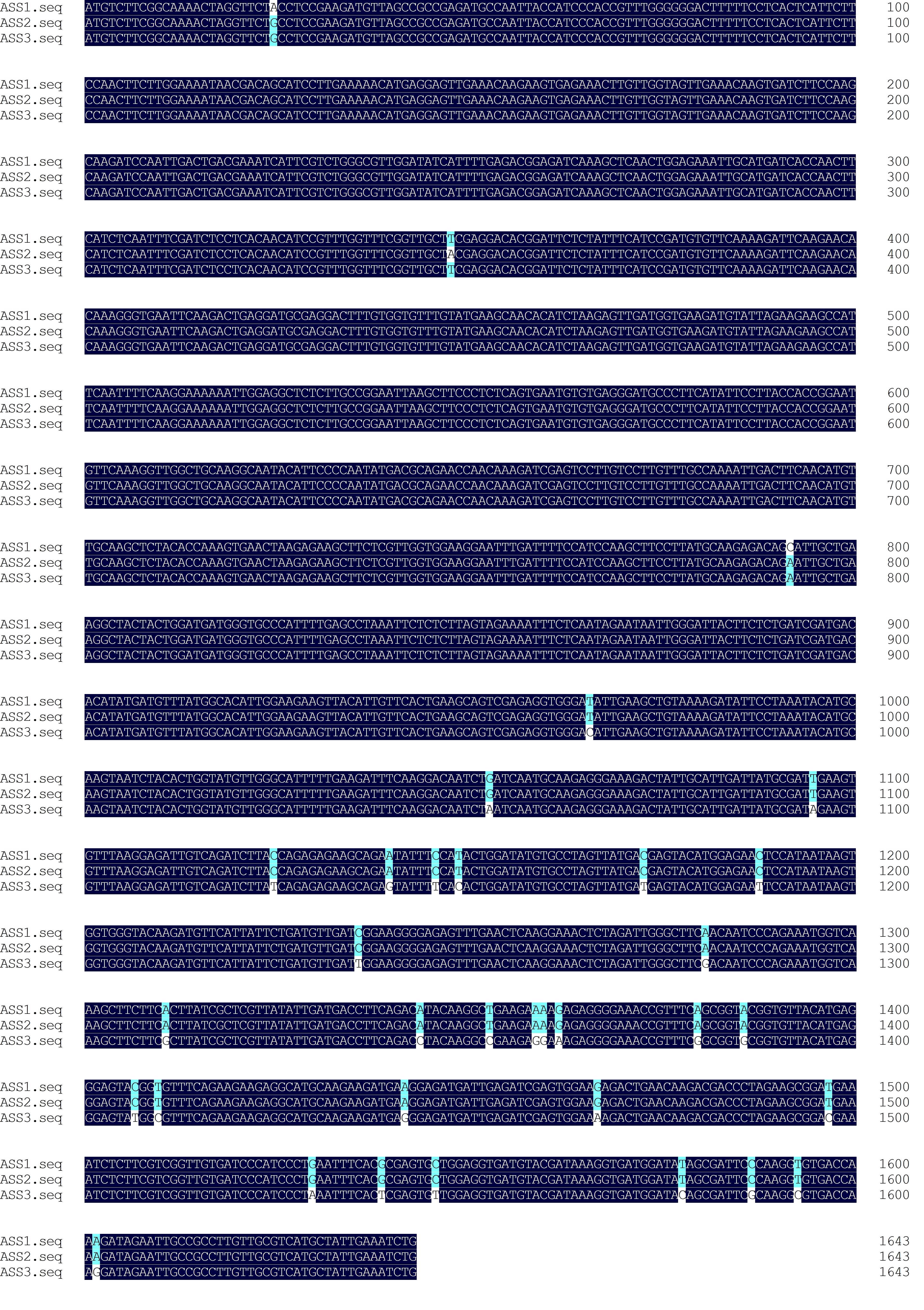

Supplement: Additional file 7: Figure S5 — Alignment of nucleotide sequences for 3 sesquiterpene synthase (ASS) genes. [file 1471-2164-14-227-S7.docx]
