# Supplementary material for: Identification of genes related to agarwood formation: transcriptome analysis of healthy and wounded tissues of Aquilaria sinensis
Source: BMC Genomics. 2013 Apr 8;14:227. doi: 10.1186/1471-2164-14-227 (PMC3635961; doi:10.1186/1471-2164-14-227)

**Additional file 8: Figure S6.** Alignment of deduced amino acid sequences for 3 ASSs. Residues shaded in black are conserved among the three sequences. The RRx8W motif at the N-terminus is indicated with red box, and the DDxxD motif known to be a divalent metal ion substrate-binding site is indicated with green box.


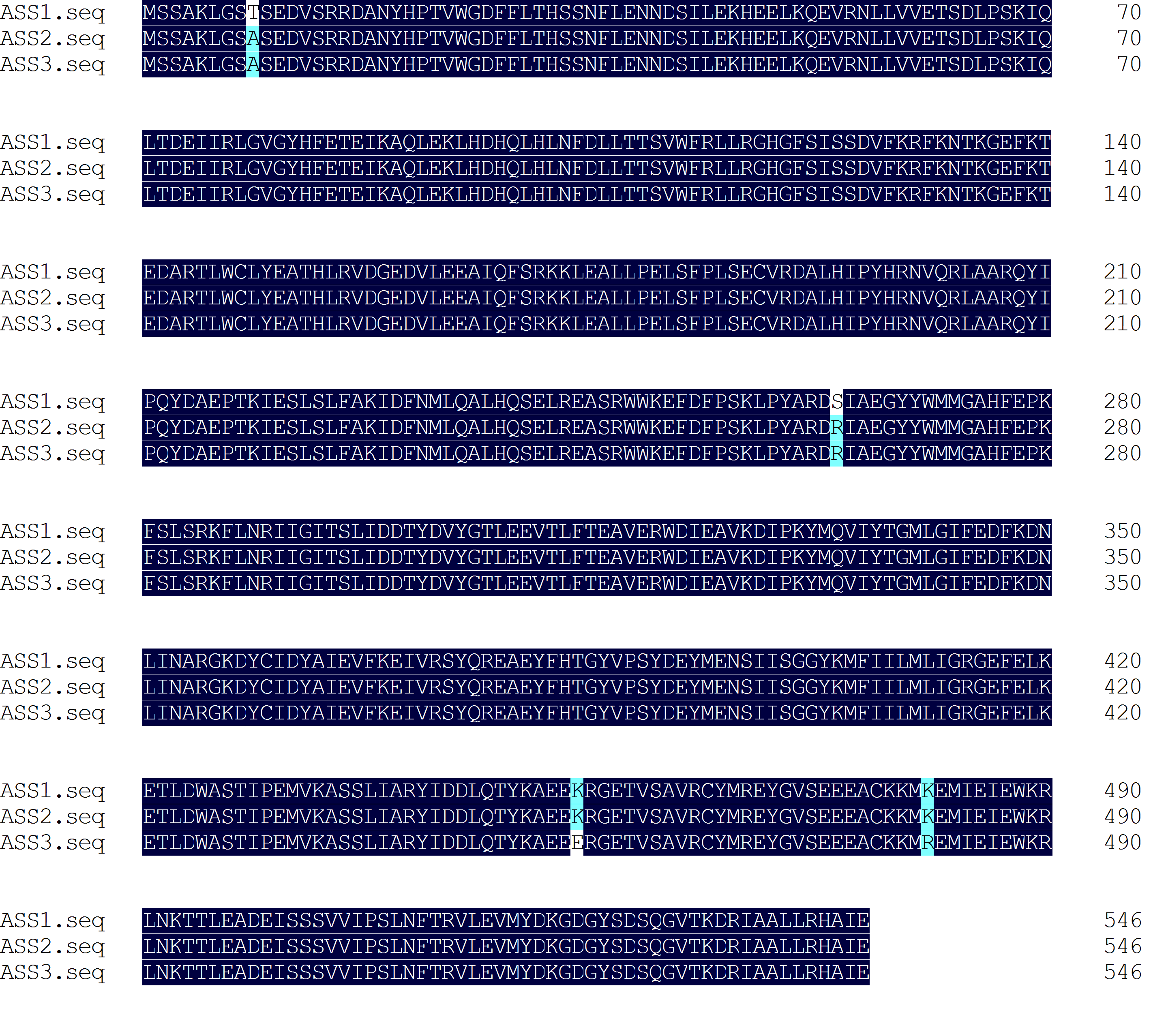

Supplement: Additional file 8: Figure S6 — Alignment of deduced amino acid sequences for 3 ASSs. [file 1471-2164-14-227-S8.docx]
