# Supplementary material for: Identification of genes related to agarwood formation: transcriptome analysis of healthy and wounded tissues of Aquilaria sinensis
Source: BMC Genomics. 2013 Apr 8;14:227. doi: 10.1186/1471-2164-14-227 (PMC3635961; doi:10.1186/1471-2164-14-227)

**Additional file 9: Figure S7. Phenetic analysis of translated full length sesquiterpene synthase sequences (*ASS1-3*) from *A. sinensis* and some characterized sesquiterpene synthase sequences from other plant species.** Amino acid sequences were aligned using the CLUSTALW program, and evolutionary distances were computed using MEGA4 with the Poisson correction method. Bootstrap values obtained after 1,000 replications are indicated on the branches. Note that: ACY37194.1, ACY37195.1, ACY37196.1, ACY37197.1 are corresponding with AcC1, AcC2, AcC3, AcC4 in the article, respectively.


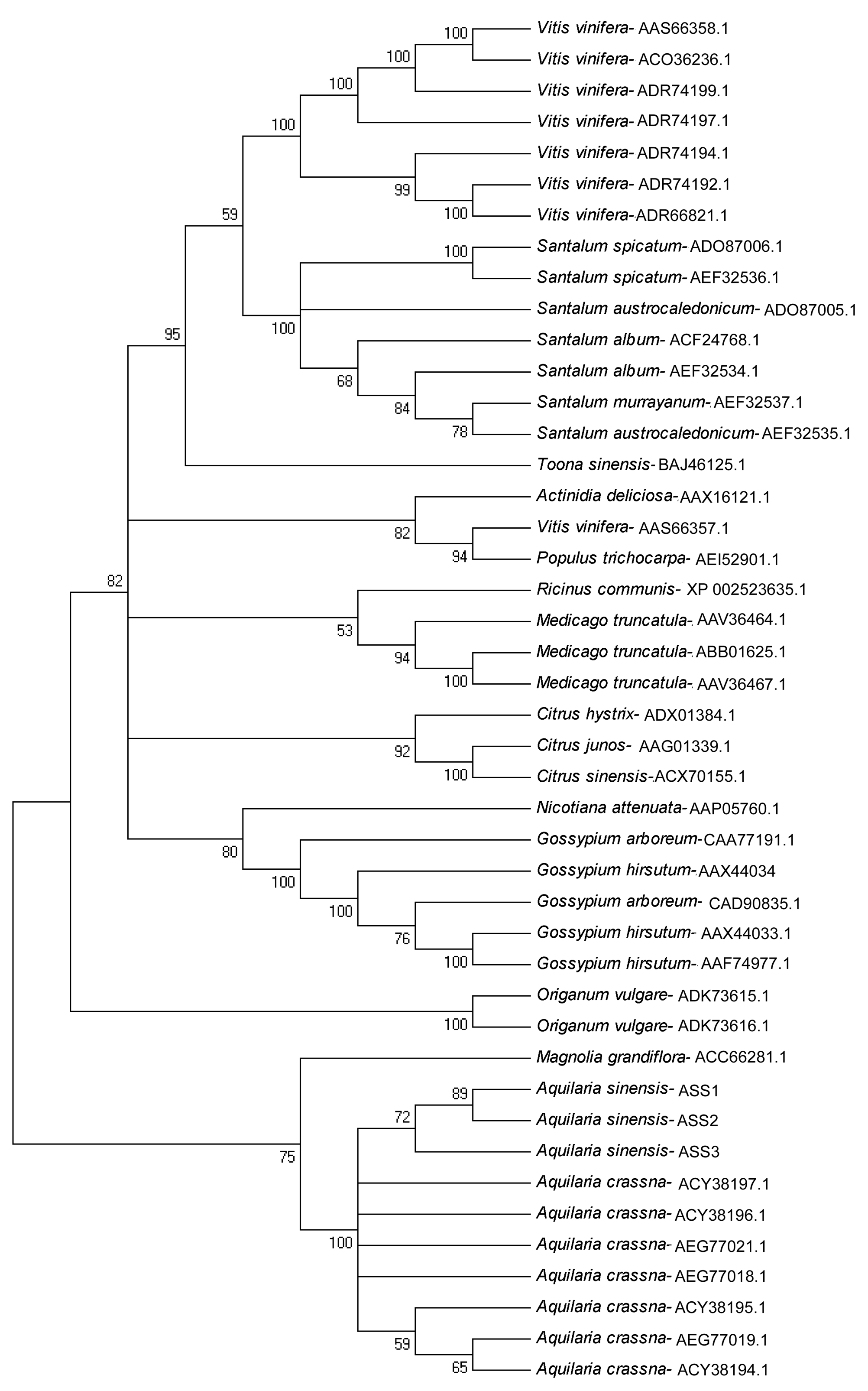

Supplement: Additional file 9: Figure S7 — Phenetic analysis of 3 full-length sesquiterpene synthase sequences from A. sinensis (ASS1-3) and some characterized sesquiterpene synthase sequences from other plant species. Bootstrap values after 1000 replications are shown on the branches. [file 1471-2164-14-227-S9.docx]
