# Supplementary material for: Identification of genes related to agarwood formation: transcriptome analysis of healthy and wounded tissues of Aquilaria sinensis
Source: BMC Genomics. 2013 Apr 8;14:227. doi: 10.1186/1471-2164-14-227 (PMC3635961; doi:10.1186/1471-2164-14-227)
Supplement: Additional file 10: Table S3 — Major transcription factors identified from A. sinensis. [file 1471-2164-14-227-S10.docx]

**Additional file 10: Table S3. Major transcription factors identified from *A. sinensis*.**

| **Class of transcription factors** | **Numbers of unigenes** | **Numbers of 454 reads** | |
| --- | --- | --- | --- |
|  |  | **Healthy** | **Wounded** |
| ABI3VP1 | 182 | 1019 | 1381 |
| AP2-EREBP | 86 | 1663 | 867 |
| ARF | 27 | 134 | 234 |
| ARR-B | 10 | 30 | 62 |
| Alfin-like | 6 | 90 | 39 |
| BBR/BPC | 4 | 31 | 25 |
| BES1 | 10 | 72 | 137 |
| BSD | 13 | 86 | 106 |
| C2C2-CO-like | 7 | 16 | 15 |
| C2C2-Dof | 15 | 42 | 89 |
| C2C2-GATA | 26 | 349 | 181 |
| C2H2 | 150 | 1721 | 1652 |
| C3H | 194 | 2214 | 1921 |
| CAMTA | 21 | 66 | 49 |
| CCAAT | 25 | 332 | 219 |
| CPP | 5 | 4 | 7 |
| CSD | 8 | 804 | 30 |
| DBP | 31 | 245 | 246 |
| E2F-DP | 4 | 6 | 3 |
| EIL | 5 | 99 | 97 |
| FAR1 | 2345 | 18077 | 21058 |
| FHA | 63 | 1009 | 610 |
| G2-like | 31 | 228 | 127 |
| GRAS | 72 | 661 | 812 |
| GRF | 18 | 53 | 103 |
| GeBP | 7 | 80 | 62 |
| HB | 88 | 488 | 525 |
| HRT | 3 | 23 | 37 |
| HSF | 15 | 132 | 100 |
| LIM | 5 | 18 | 10 |
| LOB | 17 | 40 | 54 |
| MADS | 189 | 1538 | 1327 |
| MYB | 108 | 1454 | 1639 |
| MYB-related | 150 | 1110 | 1187 |
| NAC | 166 | 1313 | 1187 |
| OFP | 5 | 14 | 15 |
| Orphans | 72 | 414 | 403 |
| PBF-2-like | 5 | 18 | 15 |
| PLATZ | 4 | 30 | 76 |
| RWP-RK | 63 | 178 | 171 |
| S1Fa-like | 2 | 60 | 35 |
| SBP | 31 | 366 | 117 |
| Sigma70-like | 7 | 64 | 26 |
| TAZ | 7 | 36 | 33 |
| TCP | 12 | 16 | 32 |
| TIG | 16 | 74 | 58 |
| TUB | 11 | 131 | 121 |
| Tify | 29 | 553 | 111 |
| Trihelix | 17 | 108 | 110 |
| ULT | 3 | 15 | 6 |
| VOZ | 4 | 36 | 30 |
| WRKY | 99 | 777 | 521 |
| bHLH | 169 | 907 | 758 |
| bZIP | 66 | 498 | 449 |
| mTERF | 56 | 576 | 455 |
| zf-HD4 | 2 | 3 | 4 |
