# Supplementary material for: Identification of genes related to agarwood formation: transcriptome analysis of healthy and wounded tissues of Aquilaria sinensis
Source: BMC Genomics. 2013 Apr 8;14:227. doi: 10.1186/1471-2164-14-227 (PMC3635961; doi:10.1186/1471-2164-14-227)
Supplement: Additional file 11: Table S4 — Unigenes annotated as being related to the calcium signaling and mitogen-activated protein kinase signaling pathways. [file 1471-2164-14-227-S11.docx]

**Additional file 11: Table S4. Unigenes annotated as relating to calcium signaling pathway and MAPK signaling pathway based on KEGG calssification.**

| **Pathway** | **No. of unigenes** | | **Unigenes** |
| --- | --- | --- | --- |
| Calcium signaling | 25 | Cluster46113;Cluster51765.seq.Contig1;Cluster53146.seq.Contig1; Cluster53146.seq.Contig2;Cluster58037.seq.Contig1;Cluster35047; Cluster60846.seq.Contig;Cluster38563;Cluster38646;Cluster40982; Cluster51070.seq.Contig1; Cluster54428.seq.Contig1; Cluster2504;  Cluster55086.seq.Contig1; Cluster58725.seq.Contig3; Cluster857;  Cluster60029.seq.Contig1; Cluster60846.seq.Contig1; Cluster19851;  Cluster25015; Cluster42252;Cluster43899;Cluster46933;  Cluster47159; Cluster47381; | |
| MAPK signaling | 41 | Cluster60663.seq.Contig4; Cluster51135.seq.Contig2;Cluster21310;  Cluster52721.seq.Contig1; Cluster53146.seq.Contig1; Cluster22861;  Cluster53146.seq.Contig2; Cluster56959.seq.Contig1; Cluster36621;  Cluster57659.seq.Contig1; Cluster59667.seq.Contig1; Cluster26315;  Cluster61579.seq.Contig1; Cluster61928.seq.Contig1; Cluster36629;  Cluster62712.seq.Contig1; Cluster62975.seq.Contig2; Cluster47381;  Cluster62975.seq.Contig3; Cluster63504.seq.Contig1; Cluster44431;  Cluster63504.seq.Contig2; Cluster63793.seq.Contig2; Cluster49916;  Cluster53700.seq.Contig1; Cluster54633.seq.Contig1; Cluster17888;  Cluster58365.seq.Contig1;Cluster58365.seq.Contig2;Cluster18962;  Cluster59667.seq.Contig2; Cluster60029.seq.Contig1; Cluster19490;  Cluster60234.seq.Contig1;Cluster61508.seq.Contig1; Cluster14946;  Cluster62975.seq.Contig1; Cluster63793.seq.Contig1; Cluster177;  Cluster60663.seq.Contig1; Cluster60663.seq.Contig3; | |
