# Supplementary material for: Identification of genes related to agarwood formation: transcriptome analysis of healthy and wounded tissues of Aquilaria sinensis
Source: BMC Genomics. 2013 Apr 8;14:227. doi: 10.1186/1471-2164-14-227 (PMC3635961; doi:10.1186/1471-2164-14-227)
Supplement: Additional file 13: Table S6 — Primers used in this study. [file 1471-2164-14-227-S13.docx]

**Additional file 13: Table S6. Primers used in this study.**

**1. Primers for cloning ORF cDNA of *AsFPS* and *ASS1-3*.**

*AsFPS:*

forward primer: 5'-ATGGCGGATCTCAGATCGAC-3'

reverse primer: 5'-CTTCTGCCTCTTGTAGATTTTCCC-3'

*ASS1-3:*

forward primer: 5'-ATGGTCGACCTGCAGGCG-3'

reverse primer: 5'-TCAGATTTCAATAGCATGACGCAAC-3'

**2. Gene-specific primers for real-time PCR analysis of gene expression**

*Internal Control: AcHistone*

forward primer: 5'-GTACCGCTACCGGAGGGAAGTTGAAGA-3'

reverse primer: 5'-CTTCTTGGGCGACTTGGTAGCCTTGGT-3'

*ASS1:*

forward primer: 5'-AAGAAGATGAAGGAGATGATTGAGA-3'

reverse primer: 5'-TAGATACTCAAGCTATGCATCCAAC-3'

*ASS2:*

forward primer: 5'-AAGAAGATGAAGGAGATGATTGAGA-3'

reverse primer: 5'-TTTCAATAGCATGACGCAACAAG-3'

*Cluster61437.seq.Contig2 (DXPS):*

forward primer: 5'-CTCTTGAGATTGGCAAAGGCAG-3'

reverse primer: 5'-ATTGCTATTGGTCGCACCTTTAC-3'

*Cluster60873.seq.Contig1 (HMGR):*

forward primer: 5'-CGTTCTATTGAGAGATGGGATGAC-3'

reverse primer: 5'-ATGGCATCACCAGTGCTACAAG-3'

*Cluster58892.seq.Contig1 (AsFPS):*

forward primer: 5'-ATGATAACTACGGGAAGGATAACGC-3'

reverse primer: 5'-ACTTGTCCACCATTCCGTCGC-3'

*Cluster57215.seq.Contig1 (CDPK1):*

forward primer: 5'-CCATTTCTAAAGATAGGCTTGTGAC-3'

reverse primer: 5'-AAAATCCTCAACTTCTTCAATCAAG-3'

*Cluster58835.seq.Contig1 (CDPK2):*

forward primer: 5'-CGGGGAGAGTTCGGTATTACTTATC-3'

reverse primer: 5'- GTTACCAAGACCATTGTGGAAGTTG-3'

*Cluster53662.seq.Contig1 (CDPK3):*

forward primer: 5'-GGATGATAGAGTCGCCCCAG-3'

reverse primer: 5'- TAAATAAGACGGAGAGAGAGGAAAA-3'

*Cluster60806.seq.Contig3 (CDPK5):*

forward primer: 5'-GGTCTAAAAGAAATGTTCAAGGCAA-3'

reverse primer: 5'- GTGAGGAACATCTTGTAGCAGCAT-3'

*Cluster51236.seq.Contig1 (CDPK6):*

forward primer: 5'-GTCATTACAGTGAGCGAGCGG-3'

reverse primer: 5'- GTGATACTTTACATTTTGCTGTGCG-3'

*Cluster55740.seq.Contig1 (ERF115):*

forward primer: 5'-ATGGGGAAAGTGGGCGGC-3'

reverse primer: 5'- ACCTGCCGGGTAGTACTGAGCC-3'

*Cluster51020.seq.Contig1 (EBF):*

forward primer: 5'-CAAAGCAGCCACATCACTTGAGAAC-3'

reverse primer: 5'- TACCAGCCTCAATACCTTCAGTCAA-3'

*Cluster61431.seq.Contig2 (EIN3):*

forward primer: 5'-TCTGACAATCTACGGGAGTGGTG-3'

reverse primer: 5'- ATGGTGGTGGCACTCCTTTCTC-3'

*Cluster61879.seq.Contig1 (ETR1):*

forward primer: 5'-GATGTGATTGGTGATGAGAAACG-3'

reverse primer: 5'- AAAGATTTGGAATATCCTGTGGG-3'

*Cluster64407.seq.Contig2 (ACO3):*

forward primer: 5'-CATCTTGCTCTTCCAGGACGAC-3'

reverse primer: 5'- CTGTTTGTTCTCCTCCGCCA-3'

*Cluster61772.seq.Contig1 (AOSC):*

forward primer: 5'-TTCAACTGGCTCCCCTGCT-3'

reverse primer: 5'- TGTTCGGGAAGAAGAGTTTCATGC-3'

*Cluster58214.seq.Contig1 (JAip):*

forward primer: 5'-TCTCCAAGGTAGCTCTCGACATG-3'

reverse primer: 5'- GGAGGGAACGATCGCCG-3'

*Cluster52765.seq.Contig1 (noxB):*

forward primer: 5'-GCCGTCTACACATTCCCGA-3'

reverse primer: 5'- CCGGGTTGCAATTCTTTCA-3'

*Cluster63395.seq.Contig1 (APX1):*

forward primer: 5'-AAGGCTTACCCGACTGTGAG-3'

reverse primer: 5'- AGTAACCTCAACTGCCACGAC-3'

*Cluster58923.seq.Contig1 (SODCP):*

forward primer: 5'-TTTTCCGTGAAGTTCCTCCG-3'

reverse primer: 5'- CGGATTGAAATGTGCTCCAGTT-3'

*Cluster59297.seq.Contig1 (SODA):*

forward primer: 5'-GAAGGAGGTGGTGAGCCGC-3'

reverse primer: 5'- TCTTCAAGTAGTCAGGTCTAACATT-3'

*Cluster57933.seq.Contig1 (WRKY2):*

forward primer: 5'-AAAAGATTCTGGGTTCAAGCAAAG-3'

reverse primer: 5'- CGAGGATTGGAGGTTGAAGGG-3'

*Cluster58457.seq.Contig1 (WRKY4):*

forward primer: 5'-TCTCGCCTTCCTCTTACTTCG-3'

reverse primer: 5'- ATCCGATTGGAACCCGTTG-3'

*Cluster25441 (WRKY23):*

forward primer: 5'-TGGTGGCATCACTTTGGGT-3'

reverse primer: 5'- GATTTACCTGCCTTTGGTGAACTG-3'

*Cluster54069 (WRKY32):*

forward primer: 5'-GGCAGGCTCGTCCTTGTTC-3'

reverse primer: 5'- GATCTCGCTCCACGTGTTTCC-3'

*Cluster31496 (WRKY60):*

forward primer: 5'- ACAGATTGGTCATAACATGAACTCG-3'

forward primer: 5'-CTTCAGTTCAGCAGAGACTTCAGCC-3'

*Cluster17738 (MYBJI):*

forward primer: 5'-TTTTGGTGGGGTTAGGCTCTT-3'

reverse primer: 5'- TCGTCACAAAGTTCCTGGAGATG-3'

*Cluster56958.seq.Contig1 (APL1):*

forward primer: 5'-CAAAAGGTGTTCTTAGAGTTATGGG-3'

reverse primer: 5'- CCCTTGAGCCTCTATCCGC-3'

*Cluster58257.seq.Contig1 (APL2):*

forward primer: 5'-CCTTCACCTTTCATTGGAGCC-3'

reverse primer: 5'- TCAGATAAATCAACAGCAGAAGCC-3'

*Cluster51480.seq.Contig1 (MYB4):*

forward primer: 5'-GATAAAGAACCACTGGCACACCC-3'

reverse primer: 5'- CAGCGTGCTCCAGGGGG-3'

*Cluster54321.seq.Contig1 (MAPK5):*

forward primer: 5'-AGTGACCCTACCGTGAGGAGAC-3'

reverse primer: 5'- AATTTGAGGGTTTAATATCGCGG-3'

*Cluster60618.seq.Contig1 (MAPK15):*

forward primer: 5'-TCGCAGAGATGCTTACAGGAAAAC-3'

reverse primer: 5'- ATGCGTTCAAGAAGACGAAGAGC-3'

*Cluster22126 (MAPK2):*

forward primer: 5'-CGGCAAGCAAACAGGGG-3'

reverse primer: 5'- TATGGTCGCTCGGCTGTATCG-3'

*Cluster59667.seq.Contig1 (MAPK3):*

forward primer: 5'-GCTATTGCTGAACTCCTCGGAT-3'

reverse primer: 5'- CAATGGCTGACGAGGATGTG-3'

*Cluster57968.seq.Contig1 (MPKK2):*

forward primer: 5'- ATGCTCTGCCCTTCGTCAAC-3'

reverse primer: 5'- GCAAAGGAGCAGAGTGGGATAG-3'
